# From Dogs to Robots: Pet-Assisted Interventions for Depression in Older Adults—A Network Meta-Analysis of Randomized Controlled Trials

**DOI:** 10.3390/healthcare14010038

**Published:** 2025-12-23

**Authors:** Mei-Ling Dai, Berne Ting, Ray Jui-Hung Tseng, Yu-Ling Huang, Chia-Ching Lin, Min-Hsiung Chen, Pan-Yen Lin, Tzu-Yu Liu

**Affiliations:** 1 Department of Nursing, Wei Gong Memorial Hospital, Miaoli 351498, Taiwan; 045064@tool.caaumed.org.tw; 2 Department of Nursing, Yuanpei University of Medical Technology, Hsinchu 30015, Taiwan; 3 Center for General Education, Hungkuang University, Taichung 433304, Taiwan; berne.ting@uspace.hk.edu.tw; 4 School of Medicine, College of Medicine, National Cheng Kung University, Tainan 701401, Taiwan; i54131046@gs.ncku.edu.tw; 5 Department of Medical Quality, Teaching and Research Section, Wei Gong Memorial Hospital, Miaoli 351498, Taiwan; 045870@tool.caaumed.org.tw; 6 Department of Healthcare Management, Yuanpei University of Medical Technology, Hsinchu 30015, Taiwan; 7 Department of Occupational Therapy, Wei Gong Memorial Hospital, Miaoli 351498, Taiwan; 045354@tool.caaumed.org.tw; 8 Department of Neurosurgery, Wei Gong Memorial Hospital, Miaoli 351498, Taiwan; 093178@tool.caaumed.org.tw; 9 Mind-Body Interface Laboratory (MBI-Lab), China Medical University, Taichung 404328, Taiwan; 10 Department of Psychiatry, Wei Gong Memorial Hospital, Miaoli 351498, Taiwan; 11 Department of Nursing, Hsin-Sheng College of Medical Care and Management, Taoyuan 32544, Taiwan

**Keywords:** animal-assisted therapy, robotic pets, late-life depression, network meta-analysis, older adults

## Abstract

**Background/Objectives**: Late-life depression is prevalent yet frequently underdiagnosed, underscoring the need for accessible and safe non-pharmacological approaches. Pet-assisted interventions, including live animal-assisted therapy and robotic pets, have gained attention, but their comparative effectiveness remains unclear. This study aimed to evaluate and rank different pet-assisted approaches for reducing depressive symptoms in older adults using network meta-analysis. **Methods**: We systematically searched PubMed, Embase, Web of Science, and the Cochrane Library up to August 2025 for randomized controlled trials involving adults aged 60 years or older with depression. The protocol was prospectively registered on INPLASY (INPLASY2025100023). Depression severity, assessed using validated scales, was synthesized using a frequentist random-effects network meta-analysis framework. **Results**: Twenty trials involving 1073 participants were included. Live animal-assisted therapy produced the greatest reduction in depressive symptoms versus passive control (SMD −2.04; 95% CI −3.03 to −1.04). Combining it with gait training (structured walking-based activity conducted with the animal) was associated with a reduction in depressive symptoms (SMD −4.82; 95% CI −6.69 to −2.95). Robotic pets showed a directionally beneficial but non-significant effect (SMD −1.21; 95% CI −2.79 to 0.38). **Conclusions**: Pet-assisted interventions are effective in reducing depressive symptoms among older adults. Live animal-assisted therapy, particularly when delivered in structured or combined formats, shows the greater benefit. Robotic pets may serve as a practical alternative when live animals cannot be implemented.

## 1. Introduction

Late-life depression is highly prevalent in long-term care institutions yet often remains overlooked. Multimorbidity, cognitive impairment, and chronic pain frequently obscure depressive symptoms, leading to underdiagnosis and delayed treatment [1,2]. Without timely intervention, depression not only accelerates functional decline but also perpetuates a vicious cycle of deteriorating health and quality of life [3,4]. Against this backdrop, pet-assisted interventions have increasingly gained attention as non-pharmacological strategies that provide both emotional support and psychosocial stimulation [5,6,7].

Growing empirical evidence suggests that both live animals and robotic pets may help alleviate depressive symptoms in older adults [8,9], although prior findings are not entirely consistent. Randomized controlled trials have reported reductions in depression scale scores, with particularly notable improvements among individuals with higher baseline severity [6,10,11]. Beyond symptom relief, pet interactions promote meaningful engagement, enhance social participation, and foster responsibility and self-efficacy, directly addressing common barriers to managing late-life depression such as social withdrawal, anhedonia, and treatment resistance [12,13].

In recent years, advances in artificial intelligence and robotics have introduced robotic pets as emerging alternatives to live animals in therapeutic contexts [14]. Prior meta-analyses suggest that live animal-assisted therapy may alleviate depressive symptoms in older adults, whereas evidence for robotic pets such as the PARO seal remains mixed [9]. Unlike live animal-assisted therapy, which involves a bidirectional human–animal relationship characterized by physiological feedback and emotional attunement, robotic-pet interventions entail human–machine interaction that lacks biological reciprocity [15,16]. Although both modalities are increasingly used in geriatric care, their relative effects have not been evaluated within a unified network meta-analytic framework that permits indirect comparisons, treatment ranking, and examination of modality- and content-specific effects. Clarifying these relationships is clinically relevant, since robotic pets offer practical advantages in feasibility, safety, and reduced caregiving burden in institutional settings [17,18].

The present study addresses this gap by conducting a network meta-analysis (NMA) to evaluate the effects of pet-assisted interventions on depressive symptoms in older adults. Unlike previous meta-analyses that examined single modalities, NMA enables simultaneous synthesis and ranking of diverse intervention types [19,20]. We hypothesized that older adults receiving pet-assisted interventions would experience significant reductions in depressive symptoms, with secondary benefits in social interaction and life satisfaction. Ultimately, this study aims to clarify whether robotic pets can serve as effective alternatives to live animals, thereby advancing evidence-based approaches in geriatric mental health care.

## 2. Materials and Methods

This study was conducted in accordance with the Preferred Reporting Items for Systematic Reviews and Meta-Analyses guidelines, with a particular emphasis on extensions for network meta-analyses (PRISMA-NMA) [21]. The study protocol was prospectively registered in the International Platform of Registered Systematic Review and Meta-Analysis Protocols (INPLASY; registration number INPLASY2025100023).

### 2.1. Database Search and Identification

**Figure 1:**
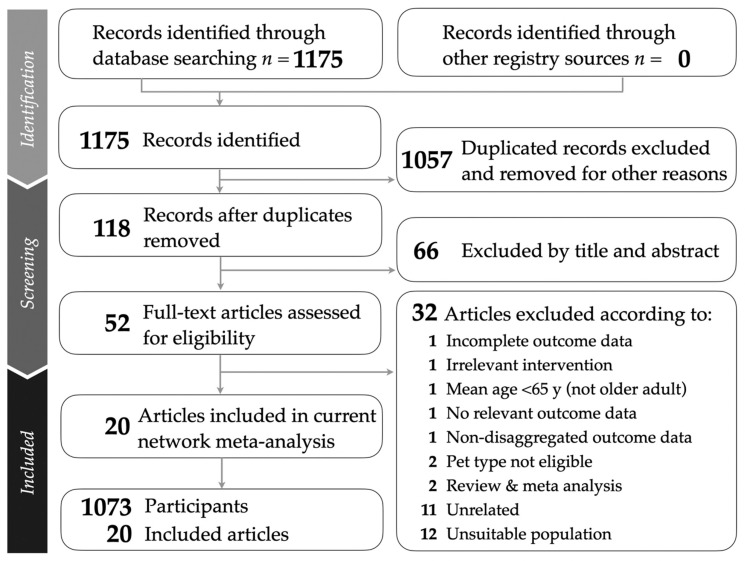
PRISMA flow diagram of the study selection and screening process for the network meta-analysis on pet-assisted interventions for depression in older adults.

A comprehensive systematic search was conducted in four electronic databases: PubMed, Web of Science, Embase, and the Cochrane Library to identify eligible studies on animal-assisted therapy for depression in older adults. The search included all publications available up to August 2025 and employed Boolean operators in combination with relevant keywords, including “pet-assisted therapy,” “animal-assisted therapy,” “animal-assisted intervention,” “robotic pet therapy,” “elderly depression,” “late-life depression,” “geriatric depression,” and “randomized controlled trials.” Both free-text terms and Medical Subject Headings (MeSH) were used, where applicable, to maximize search sensitivity. Duplicate records were removed, and clearly irrelevant studies were excluded. In addition, a manual search of the reference lists of relevant systematic reviews and meta-analyses was performed to identify potential additional studies. Two authors (Dai and Ting) independently screened the titles and abstracts of all retrieved records for eligibility. Any disagreements were resolved through discussion with a third author (Lin) until consensus was reached. This systematic and multi-step process ensured that only studies meeting the predefined inclusion criteria were included. The overall process of study identification, screening, eligibility assessment, and final inclusion is summarized in a PRISMA 2020 flow diagram (Figure 1).

### 2.2. Inclusion and Exclusion Criteria

This network meta-analysis (NMA) followed the PICO framework. We included randomized controlled trials (RCTs) that investigated the effects of animal-assisted therapy (AAT) on depression in older adults aged 60 years or above. Eligible interventions comprised either single-form AAT, including live dog-assisted therapy or robotic animal-assisted therapy (e.g., PARO robotic seal), or predefined multimodal AAT programs in which AAT was intentionally integrated with structured non-pharmacological components, such as gait training, integrated emotional–psychosocial therapy (IEPT), or reality orientation training (ROT). Control conditions included usual care, waitlist, no treatment, placebo-like activities, or other non-pharmacological interventions not involving animals. The primary outcome was depression severity assessed with validated instruments, such as the Geriatric Depression Scale (GDS), Hamilton Depression Rating Scale (HDRS), or the Cornell Scale for Depression in Dementia (CSDD).

Studies were excluded if the intervention arm involved AAT delivered only as an adjunct to pharmacological treatment or conventional psychotherapy (e.g., standard cognitive behavioral therapy, medication), if the animal species were other than dogs (e.g., cats, birds, insects, or other non-canine animals), if the comparator group also received any animal-assisted or pet-based intervention, or if quantitative outcome data were unavailable for synthesis. We also excluded non-original reports, including protocols, reviews, case reports, conference abstracts, letters, or preliminary pilot studies without complete results. Only full-text articles meeting these criteria were included in the final NMA.

### 2.3. Model Construction for Network Meta-Analysis

**Figure 2:**
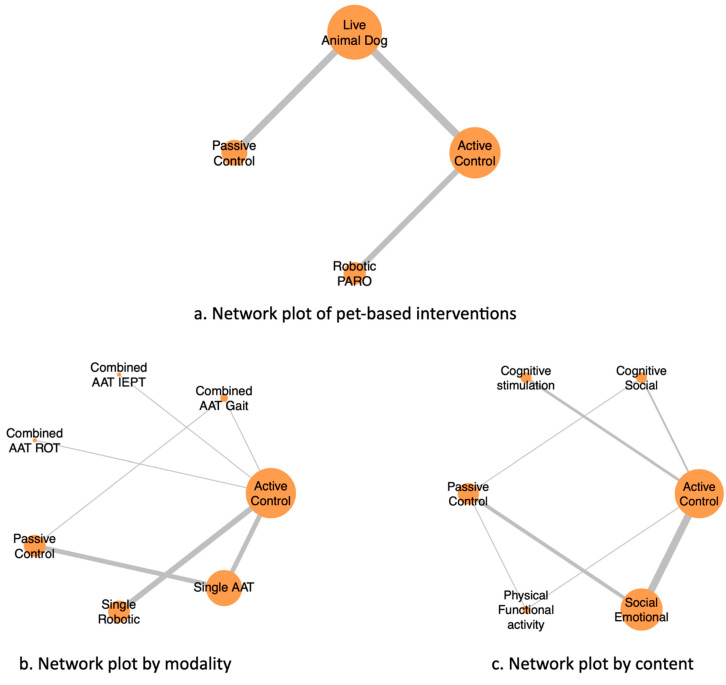
Network plots of pet-assisted interventions for depression in older adults: (**a**) pet-based interventions, (**b**) interventions classified by modality, and (**c**) interventions classified by content. Node size reflects the number of included trials; line thickness reflects the number of direct comparisons.

This study was conducted on a single evidence base and constructed three complementary network models using different intervention classification frameworks by pet type, modality, and content, to explore heterogeneity and ensure clinical transitivity (Figure 2). Node size reflects the number of included trials, and edge thickness represents the number of direct head-to-head comparisons. Different classification strategies may lead to distinct network geometries and indirect comparison structures, potentially influencing effect estimation and consistency assessment [22]. The categorization of interventions was determined through discussion between two authors (Ting and Tseng) regarding the actual components of the AAT protocols; any disagreements were resolved through consensus with a third author (Lin).

### 2.4. Risk of Bias Assessment

The methodological quality of the included studies was assessed using the Cochrane Collaboration’s Risk of Bias tool for randomized trials (RoB 2, version 2, London, UK) [23]. This instrument examines several domains of potential bias, including the randomization process, adherence to assigned interventions, management of missing outcome data, accuracy of outcome measurement, risk of selective reporting, and the overall judgment of bias.

### 2.5. Primary Outcome: Improvement of Depression in Older Adults

The primary outcome of this NMA was the improvement of depressive symptoms in older adults receiving AAT, evaluated using the standardized mean difference (SMD). The Geriatric Depression Scale (GDS) was prioritized as the main assessment tool because of its established validity in measuring depressive symptoms in late life [24]. When GDS was not available, alternative validated instruments such as the Beck Depression Inventory (BDI) [25] or the Hamilton Depression Rating Scale (HAMD) [26] were considered. This structured approach ensured consistency and accuracy in evaluating depressive outcomes across the included studies.

### 2.6. Secondary Outcomes: Intervention Modalities and Content

For the secondary outcome, we evaluated whether changes in depressive symptoms differed by intervention modality and content, summarizing effects as SMD. We conducted separate random-effects NMA for each stratification. In the modality analysis, nodes were prespecified as Single AAT, Single Robotic, and three combined formats in which AAT was integrated with another structured therapy: Combined AAT + IEPT, Combined AAT + ROT, and Combined AAT + Gait. In parallel, the content analysis used the prespecified taxonomy of Physical Functional Activity, Cognitive–Social, Social–Emotional, and Cognitive Stimulation. Both Passive Control and Active Control were retained as comparators, and primary contrasts were reported versus Passive Control.

### 2.7. Data Extraction, Handling, and Transformation

The data extraction process involved collecting participants’ demographic information, study design characteristics, details of the AAT interventions, and outcome measures. When essential data were not available in the published reports, we attempted to obtain the missing information by contacting the study authors. Data management procedures followed the recommendations of the Cochrane Handbook and were guided by established practices in medical research [27,28,29,30]. To maintain consistency, data were extracted independently by two authors, and any discrepancies were resolved through discussion; if disagreement persisted, a third author was consulted to reach consensus. The assumption of transitivity was considered reasonable, as all included populations consisted of older adults with depressive symptoms assessed using validated scales, and the potential effect modifiers—such as mean age, cognitive status, and intervention duration—were comparable across trials.

### 2.8. Statistical Analysis

To address heterogeneity across different forms of AAT, a random-effects model was applied [31]. Analyses were conducted using frequentist methods with MetaInsight (version 6.4.0; Complex Reviews Support Unit, funded by the National Institute for Health Research, London, UK) and the netmeta package within the R-based online NMA platform [32]. Forest plots and network diagrams were first generated to display pairwise comparisons among studies. Standardized mean differences (SMDs) with 95% confidence intervals were then calculated to assess improvements in depressive symptoms, along with secondary outcomes analyzed under two classification strategies [33]. Comparative rankings of relative effectiveness were subsequently performed, and statistical inconsistency was evaluated using established methods, with two-sided *p*-values < 0.05 considered statistically significant.

### 2.9. Sensitivity Analysis Methods

Sensitivity analyses were primarily conducted for the primary outcome to assess the robustness of the main treatment comparisons and ranking. A leave-one-out (one-study removal) approach was applied, sequentially excluding each trial to evaluate its influence on the pooled estimates [21]. To further test the impact of cognitive status, we prespecified sensitivity analyses based on study-level cognitive severity: (A) excluding samples with moderate to severe dementia (MMSE < 20); and (B) restricting analyses to non-dementia samples (MMSE ≥ 24 or explicitly dementia-free) [34]. When only study-level means were available, the reported mean MMSE value was used for classification. The resulting estimates and rankings were compared with those from the primary network.

### 2.10. Assessment of Publication Bias

Potential publication bias was evaluated according to the recommendations of the Cochrane Handbook for Systematic Reviews of Interventions [27]. Funnel plots were generated for comparisons involving control groups using Comprehensive Meta-Analysis software, version 4 (Biostat, Englewood, NJ, USA). In addition, the Egger regression test was applied to detect the presence of small-study effects and potential publication bias.

## 3. Results

### 3.1. Study Identification and Network Model Construction

Our study followed the PRISMA guidelines, as illustrated in Figure 1. A total of 20 RCTs were included in the analysis [5,6,7,10,11,15,16,35,36,37,38,39,40,41,42,43,44,45,46,47]. Additional details are provided in the PRISMA-NMA checklist (Supplementary Table S1), while the database search results and screening process are summarized in Supplementary Table S2. Reasons for full-text exclusions are listed in Supplementary Table S3.

A total of 20 randomized controlled trials (RCTs) involving 1073 participants were included in this network meta-analysis. The interventions identified across these studies were categorized into three main components. The primary analysis compared two major intervention types: live animal-assisted interventions (Live Animal Dog) and robotic pet-assisted interventions (Robotic PARO), whereas the secondary analyses categorized the interventions by modality: combined animal-assisted therapy with gait training (Combined AAT + Gait), combined animal-assisted therapy with integrated emotional–psychosocial therapy (Combined AAT + IEPT), combined animal-assisted therapy with reality orientation training (Combined AAT + ROT), single animal-assisted therapy (Single AAT), and single robotic pet intervention (Single Robotic); and by content: Physical Functional Activity, Cognitive Social, Social Emotional, and Cognitive Stimulation. The resulting network models for these intervention categories are shown in Figure 2.

The general characteristics of the included studies are summarized to provide an overview of their design and methodology, including authors, publication year, and country of origin. Detailed descriptions of the study designs were provided to ensure clear understanding of the methods used, with particular attention given to the intervention and control groups, documenting key information such as sample size, mean age, cognitive assessment tools (e.g., MMSE), and the specific content of each intervention. Information regarding control strategies, including their type and concise descriptions, was also provided. In addition, data were extracted on intervention duration, frequency, session structure, and total treatment time, as well as outcome measures of depression using validated scales such as the Geriatric Depression Scale (GDS), Cornell Scale for Depression in Dementia (CSDD), and Beck Depression Inventory (BDI). The assessment results of each study are presented in Table 1.

### 3.2. Studies’ Quality and Risk of Bias Assessment

In the randomization process, 75% (15/20) of the included studies were assessed as having low risk of bias, while 20% (4/20) were rated high risk and 5% (1/20) showed some concerns. Regarding intervention adherence, 50% (10/20) of the studies demonstrated low risk and 50% (10/20) showed some concerns. Missing outcome data were well controlled, with 80% (16/20) of studies rated low risk and 20% (4/20) showing some concerns; none were categorized as high risk. The quality of outcome measurement showed greater variability: 40% (8/20) of studies had low risk, 50% (10/20) had some concerns, and 10% (2/20) were rated high risk. For selective reporting, 85% (17/20) of studies were classified as low risk and 15% (3/20) had some concerns. Overall, 25% (5/20) of studies were assessed at low risk of bias, 60% (12/20) at some concerns, and 15% (3/20) at high risk (see Figure S1). Missing outcome data and selective reporting were generally well controlled across studies. In contrast, intervention adherence and the overall risk-of-bias ratings showed greater variability. Although most studies achieved low risk in randomization, 20% were rated as high risk. Overall, 25% of studies were at low risk, 60% at some concerns, and 15% at high risk. Detailed risk evaluations for each category are provided in Table S4.

### 3.3. Main Outcome: Effectiveness of Pet-Assisted Interventions for Alleviating Depression in Older Adults

**Figure 3:**
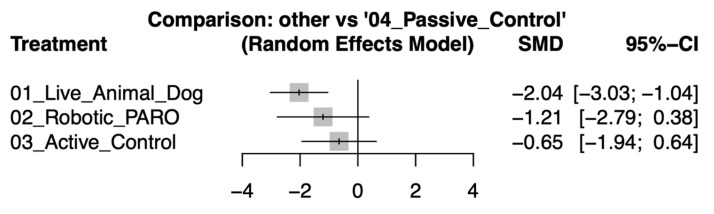
Forest plot of standardized mean differences (SMD) with 95% confidence intervals for pet-assisted interventions compared with passive control in reducing depressive symptoms among older adults.

In the primary network meta-analysis, live animal-assisted interventions showed the greatest reduction in depressive symptoms compared with passive control (SMD −2.04, 95% CI −3.03 to −1.04). Robotic pet-assisted interventions demonstrated a reduction with a wider confidence interval (SMD −1.21, 95% CI −2.79 to 0.38), while active control showed a smaller and non-significant change (SMD −0.65, 95% CI −1.94 to 0.64). The forest plot summarizing these results is shown in Figure 3. Table 2 provides a comprehensive overview of the ranking and pairwise comparisons among all intervention groups, while detailed pairwise comparisons between individual study arms are presented in Figure S2.

### 3.4. Secondary Outcomes: Network Meta-Analyses Stratified by Modality and Content

**Figure 4:**
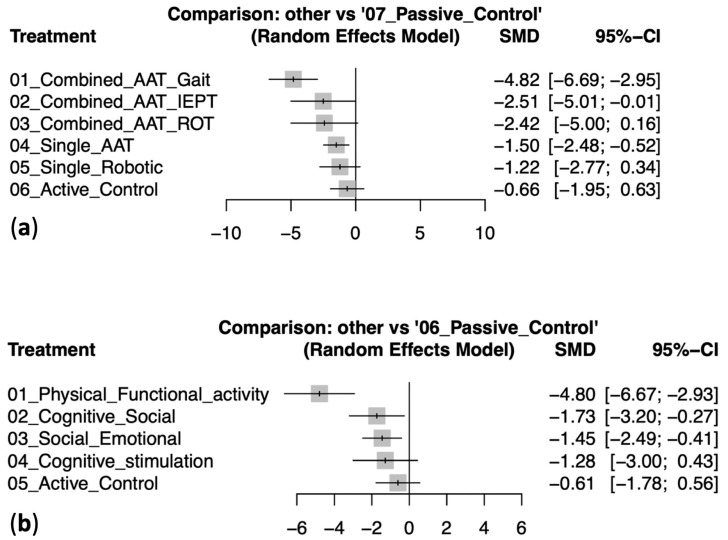
Forest plots of standardized mean differences (SMD, 95% CI) for pet-assisted interventions versus passive control in reducing depressive symptoms among older adults: (**a**) by modality and (**b**) by content.

In the modality-based network meta-analysis, Combined AAT + Gait demonstrated the greatest reduction in depressive symptoms compared with passive control (SMD −4.82, 95% CI −6.69 to −2.95), followed by Combined AAT + IEPT (SMD −2.51, 95% CI −5.01 to −0.01) and Combined AAT + ROT (SMD −2.42, 95% CI −5.00 to 0.16). Single AAT showed a moderate reduction (SMD −1.50, 95% CI −2.48 to −0.52), while Single Robotic (SMD −1.22, 95% CI −2.77 to 0.34) and Active Control (SMD −0.66, 95% CI −1.95 to 0.63) demonstrated smaller, non-significant effects. The forest plot for these results is shown in Figure 4a. Table 3a provides a comprehensive overview of the ranking and pairwise comparisons among all modality-based intervention groups.

In the content-based network meta-analysis, Physical Functional Activity showed the most pronounced improvement (SMD −4.80, 95% CI −6.67 to −2.93), followed by Cognitive Social (SMD −1.73, 95% CI −3.20 to −0.27) and Social Emotional (SMD −1.45, 95% CI −2.49 to −0.41). Cognitive Stimulation produced a smaller reduction with a wide confidence interval (SMD −1.28, 95% CI −3.00 to 0.43), and Active Control showed no significant effect (SMD −0.61, 95% CI −1.78 to 0.56). The corresponding forest plot is presented in Figure 4b. Table 3b summarizes the ranking and pairwise comparisons among all content-based intervention groups. Detailed pairwise comparisons between individual study arms for both modality- and content-based analyses are provided in Figure S3a,b.

### 3.5. Inconsistency Testing

To evaluate consistency in the NMA of pet-assisted interventions, we assessed global inconsistency using the design–by–treatment interaction model and examined local inconsistency with node-splitting analysis. For the primary NMA, the design–by–treatment model showed no statistically significant global inconsistency (*p* > 0.05), and node-splitting results indicated good agreement between direct and indirect comparisons across all treatment nodes (Table S5a). For the modality-based NMA, global inconsistency remained non-significant (*p* > 0.05), and local node-splitting analysis revealed no meaningful discrepancies between direct and indirect evidence (Table S5b). Similarly, the content-based NMA demonstrated no statistically significant inconsistency at the global level (*p* > 0.05), and node-splitting comparisons showed no evidence of local inconsistency among the included interventions (Table S5c).

### 3.6. Sensitivity Analysis

Sensitivity analyses using a leave-one-out approach showed that removal of any single study (A–T) did not materially change the pooled effect estimates or the treatment ranking. The direction and statistical significance of the primary comparisons (live animal dog, robotic PARO, and active control vs passive control) remained stable, indicating that no individual study unduly influenced the network meta-analysis results (Figure S4A–T). Additional sensitivity analyses stratified by cognitive function levels (Normal, MCI, MCI-MID, MID, MID-SEV, SEV) showed similar patterns across all strata. The overall direction and relative ranking of interventions were consistent with the main analysis, with Combined AAT + Gait showing the largest SMDs, followed by other combined AAT formats, while robotic interventions remained non-significant (Figure S4-Cog).

### 3.7. Publication Bias

Egger’s regression test for small-study effects yielded an intercept (B_0_) of −0.48 (95% CI −1.73 to 0.77; t = 0.80, df = 18) with a two-tailed *p* = 0.43 (one-tailed *p* = 0.22), suggesting no statistically significant evidence of publication bias among the included studies (Figure S5).

## 4. Discussion

### 4.1. Main Findings and Clinical Implications

This NMA synthesizing 20 RCTs with 1073 older adults found that live dog-assisted AAT produced the most robust reduction in depressive symptoms, while multimodal AAT programs, particularly those integrating gait training (AAT + Gait) or emotional–psychosocial therapy (AAT + IEPT), were more effective than single-format AAT or robotic pet (PARO) interventions. Content-based analyses further showed that programs emphasizing physical functional activity (PFA) achieved the greatest antidepressant effect. These results suggest that authentic multisensory interaction with live animals, combined with structured physical or psychosocial engagement, may enhance motivation, emotional bonding, and neurobiological recovery in late-life depression. The findings were consistent across sensitivity and inconsistency testing, and no small-study or publication bias was detected, supporting the overall robustness of the evidence.

### 4.2. Significance of Results in the Context of Current Research

Prior to our study, several systematic reviews and meta-analyses investigated the effects of animal-assisted interventions on late-life depression. Villarreal-Zegarra et al. conducted a meta-analysis synthesizing 23 RCTs that compared AAT with pet-robot interventions and reported a moderate antidepressant effect for AAT (Hedges’ g = −0.72), while finding no significant benefit for PRI; however, their analysis provided only pooled estimates without differentiating intervention modalities or contents [9]. Borgi et al. focused exclusively on dog-visiting programs across 10 trials and found a large effect on depressive symptoms (SMD = −1.00), particularly for structured and ≥10-week interventions. However, their analysis was limited to dog visits and lacked comparisons across other animal types or intervention formats [48]. Chang et al. conducted a large review of 47 studies, including live animals and some robotic pets, and reported a large pooled effect (SMD = −1.31) and broader psychosocial, behavioral, physiological, and cognitive benefits, yet with very high heterogeneity and no comparative ranking [49]. Orr et al. conducted a review that included 27 studies and were the first to contrast AAT with animal-assisted activities (AAA), finding that AAT outperformed AAA (SMD = −0.57) and that interventions ≥ 8 weeks were more effective [17]. Nonetheless, it remained a conventional pairwise meta-analysis without relative treatment ranking or network consistency evaluation.

Our NMA advances the field in several keyways. First, we not only compared live animals (particularly dogs) versus robotic pets (e.g., PARO) but also constructed a three-level classification framework (by animal type, intervention modality, and intervention content) to build a robust treatment network, providing, for the first time, a comparative ranking of intervention efficacy. We found that Combined AAT with gait training showed the greatest benefit (SMD = −4.82), followed by AAT combined with IEPT or ROT, while single AAT maintained moderate effectiveness. This granularity addresses the limitations of prior reviews that reported only pooled average effects and equips clinicians with evidence-based guidance for selecting and designing non-pharmacological interventions. Second, we offer the first quantitative evaluation of robotic pet interventions in direct comparison with live animal therapies. While Villarreal-Zegarra and Chang identified insufficient evidence for PRI, our findings demonstrate that PARO shows a directionally favorable but statistically non-significant effect (SMD = −1.21, 95% CI −2.79–0.38), offering valuable insight for care settings where live animals may not be feasible. Finally, our study applied PRISMA-NMA standards with consistency checks, leave-one-out sensitivity analyses, and publication bias testing, thus providing more methodologically rigorous and robust evidence than conventional meta-analyses. Although heterogeneity and limited sample sizes remain challenges, this NMA fills a critical gap by delivering a network-based, structured comparison and clear efficacy ranking of animal-assisted interventions for late-life depression, enabling more targeted clinical decision-making. Additionally, sensitivity analyses stratified by cognitive severity confirmed the stability of these findings across all cognitive levels—from normal cognition to severe dementia—indicating that the antidepressant effects of animal-assisted interventions are broadly applicable across diverse cognitive profiles in older adults. Overall, across the included trials, a generally consistent pattern of depressive symptom improvement was observed, despite variation in settings, populations, and intervention formats.

### 4.3. Possible Explanations for the Observed Results

The present NMA indicates that combined animal-assisted interventions tended to produce relatively greater improvements in depressive symptoms in older adults, followed by combined AAT with emotion- or cognition-focused elements, whereas robotic pets such as PARO showed a directionally beneficial but non-significant effect. These findings can be understood through a set of interconnected physiological, psychological, and social mechanisms, which collectively explain the observed antidepressant effects and highlight practical implications for clinical implementation.

From a physiological perspective, animal interaction is thought to induce neuroendocrine modulation and autonomic regulation, both of which play crucial roles in mood stabilization [15]. Gentle physical contact, eye contact, and bonding behaviors have been reported to stimulate oxytocin release and suppress cortisol and hypothalamic–pituitary–adrenal (HPA) axis hyperactivity, reducing stress and anxiety while fostering emotional calmness [50]. Neuroimaging evidence suggests that animal-assisted activities can activate the mesolimbic dopamine pathway, including the ventral tegmental area and nucleus accumbens, counteracting anhedonia and restoring motivation in depressed older adults [51]. Furthermore, these interventions have been associated with improved autonomic balance, as reflected by increases in high-frequency heart rate variability (HRV) and parasympathetic tone following therapy dog sessions, supporting stress resilience, better sleep quality, and mood regulation [15]. When AAT is combined with structured physical activity such as gait training or functional mobility tasks, it may further enhance cardiorespiratory fitness, muscle strength, and balance, reducing fear of falling and improving self-efficacy. Physical exertion has been shown to increase cerebral blood flow and brain-derived neurotrophic factor (BDNF) levels, facilitating neuroplasticity and emotional regulation [52]. This multimodal “animal interaction plus exercise” design may partly explain the superior ranking of combined AAT–gait interventions observed in our network model. In contrast, the non-significant effects of robotic pets may reflect their limited sensory realism and lack of biological reciprocity, although they remain useful alternatives in infection-controlled or institutional settings.

On a psychological level, animals offer unconditional emotional acceptance and companionship, mitigating loneliness, self-blame, and feelings of worthlessness that frequently underlie late-life depression. Even brief animal interaction has been associated with improved perceived social support and reduced scores on the Geriatric Depression Scale (GDS) [5,46]. Beyond passive presence, caring for and training animals, feeding, grooming, or guiding, provides a renewed sense of responsibility, mastery, and being needed, helping restore self-esteem in individuals whose depressive states often erode perceived personal value. Animals also function as a safe target for emotional projection and expression; older adults may disclose sadness, grief, or anxiety more freely to a nonjudgmental animal, which therapists can use to facilitate emotional processing and cognitive reframing. Interventions that integrate cognitive-behavioral or emotion-focused strategies with AAT further strengthen emotional regulation, positive thinking, and problem-solving skills. Additionally, activities such as remembering an animal’s name, habits, or commands offer cognitive stimulation, which helps maintain attention, memory, and executive functioning [6,35]. These cognitive and emotional benefits synergistically contribute to reducing depressive symptoms and sustaining engagement.

The social dimension is also fundamental to the observed outcomes. Animals act as natural social catalysts, stimulating conversation, eye contact, and shared enjoyment, even among socially withdrawn older adults [39,47]. Group-based AAT programs create a supportive micro-community, where participants interact not only with the animal but also with therapists and peers, rebuilding social networks that protect against isolation, a known risk factor for depression. Older adults often reclaim meaningful social roles such as “caregiver” or “mentor” when responsible for an animal, restoring purpose and dignity. Group settings also foster belonging and collective efficacy, with participants encouraging each other to stay engaged, which aligns with our finding of generally low dropout rates across AAT interventions. Such socially enriched environments may help sustain motivation and reduce relapse risk, adding practical value to long-term depression care.

Finally, although robotic pets such as PARO did not achieve statistical significance in our analysis, their clinical utility remains noteworthy. In long-term care facilities or hospitals where live animals are infeasible due to infection control, allergy risk, or logistical constraints, robotic pets still provide soothing tactile and auditory feedback, companionship, and low-pressure social stimulation [37,44]. Studies have shown reductions in loneliness and stress indicators, including lower cortisol levels and improved HRV, after regular PARO sessions [15,46]. While their antidepressant effect may be less robust than live animals, robotic pets represent an accessible and safe alternative that preserves key elements of emotional support and social facilitation, ensuring non-pharmacological care remains available where live AAT cannot be implemented.

Together, these physiological, psychological, and social mechanisms help explain the hierarchy of interventions identified in our NMA and provide actionable guidance for clinical practice. Programs for late-life depression may benefit from tailoring AAT intensity and format to patients’ functional status: highly interactive and physically engaging AAT (e.g., combined with gait training) for those with preserved mobility, and robotic pet-based or low-intensity AAT for those in infection-controlled or mobility-limited settings. Our findings fill a critical gap in the literature by directly comparing diverse AAT modalities and clarifying how their distinct biopsychosocial effects can inform personalized, feasible, and engaging non-pharmacological strategies for older adults with depression.

### 4.4. Study Limitations

This NMA has several limitations. The number and sample sizes of included RCTs were modest and may limit statistical power. Clinical heterogeneity existed across study populations, settings, and intervention components. Multiple depression scales were used, and although standardized mean differences allowed synthesis, clinical interpretability remains limited. In addition, participant expectations and the interactive nature of AAT and PARO interventions may contribute to non-specific or expectancy-related improvements, which should be considered when interpreting the findings. Finally, indirect comparisons rely on the transitivity assumption, which may be influenced by unmeasured differences in baseline severity or comorbidities. Sensitivity analyses indicated that the main findings remained stable despite these limitations.

## 5. Conclusions

In summary, our findings suggest that pet-assisted interventions may help reduce depressive symptoms in older adults. Live animal-assisted therapy tended to show greater improvements, particularly when delivered alongside structured therapeutic activities. Robotic pets demonstrated a directionally positive but statistically non-significant effect, indicating that their potential role requires further confirmation through larger, high-quality trials. Overall, these results support the potential value of animal-assisted therapy as a non-pharmacological option for late-life depression and provide guidance for optimizing intervention components, including consideration of robotic companions when live animals are not feasible.

## Figures and Tables

**Table 1:** Characteristics and outcomes of randomized controlled trials evaluating pet-assisted interventions for depression in older adults.

							Intervention Group			Control Group			
Authors & Year	Country	Study Design	Comparison	*n*	Age	Cognition/MMSE	Intervention Type	Modality	Content	Control Type	Control Descriptions	Duration of Intervention	Outcome
Ambrosi (2019) [5]	Italy	parallel-group RTC	AAT Control	17 14	82.6 87.1	≥19	Live Animal–Dog	Single–AAT	Social & Emotional	Passive	Usual care only	30 min/once weekly/10 weeks	GDS
An (2021) [10]	South Korea	parallel-group RTC	AAT Control	15 15	60.93 63.93	≥23	Live Animal–Dog	Combined–AAT + Gait	Physical/Functional activity	Active	Gait training only	30 min/once weekly/8 weeks	BDI-II
Baek (2020) [6]	South Korea	parallel-group RTC	AAT Control	14 14	82.3 82.1	10–19	Live Animal–Dog	Single–AAT	Social & Emotional	Passive	Routine care only	60 min/2× weekly/8 weeks	CSDD
Bono (2015) [35]	Italy	parallel-group RTC	AAT Control	12 12	82.1 78.3	16–24	Live Animal–Dog	Single–AAT	Cognitive stimulation	Passive	Usual care only	60 min/2× weekly/8 months	CSDD
Chen (2024) [15]	Taiwan	RCT	PARO Control	58 60	81.78 82.12	20.22 21.17	Robotic–PARO	Single–Robotic	Cognitive + Social	Active	Reminiscence only	30 min/once weekly/6 weeks	GDS-SF
Friedman (2015) [36]	USA	Pilot RCT	AAT Control	22 18	80.72	9–22	Live Animal–Dog	Combined–AAT + IEPT	Cognitive + Social	Passive	Usual care only	60–90 min/2× weekly/12 weeks	CSDD
Joranson (2015) [37]	Norway	Cluster RCT	PARO Control	27 26	83.9 84.1	<25	Robotic–PARO	Single–Robotic	Social & Emotional	Active	Normal activities only	30 min/2× weekly/12 weeks	CSDD
Kil (2019a) [11]	South Korea	parallel-group RTC	AAT Control	6 6	79.5	N/A	Live Animal–Dog	Single–AAT	Social & Emotional	Passive	Routine care only	50 min/once weekly/8 weeks	GDSSF-K
Kil (2019b) [38]	South Korea	parallel-group RTC	AAT Control	10 10	76.8	21.7 19.6	Live Animal–Dog	Combined–AAT + Gait	Physical/Functional activity	Active	IEPT only	90 min/once weekly/8 weeks	GDSSF-K
Liang (2017) [39]	New Zealand	Pilot RCT	PARO Control	13 11	67–98	38.5 34.9 (ACE-NZ)	Robotic–PARO	Single–Robotic	Social & Emotional	Passive	Usual care, no pet	30 min/2× weekly/8 weeks	CSDD
Majic (2013) [7]	Germany	parallel-group RTC	AAT Control	27 27	81.33 82.07	6.37 7.63	Live Animal–Dog	Single–AAT	Social & Emotional	Passive	Routine care only	≤45 min/once weekly/10 weeks	DMAS
Menna (2019) [40]	Italy	parallel-group RTC	AAT Control	11 11	N/A	15–16	Live Animal–Dog	Combined–AAT + ROT	Cognitive stimulation	Active	Leisure activity only	~60 min/once weekly/12 weeks	GDS
Moretti (2010) [41]	Italy	parallel-group RTC	AAT Control	10 11	86.5 83.0	15.3 18.3	Live Animal–Dog	Single–AAT	Social & Emotional	Passive	Usual care only	90 min/once weekly/6 weeks	GDS
Moyle (2013) [42]	Australia	Pilot crossover RCT	PARO Control	9 9	85.3	7.4	Robotic–PARO	Single–Robotic	Social & Emotional	Active	Human interaction only	45 min/3× weekly/5 weeks	GDS
Olsen (2016) [43]	Norway	parallel-group RTC	AAT Control	25 26	82.9 84.1	<25	Live Animal–Dog	Single–AAT	Social & Emotional	Passive	Routine care only	30 min/2× weekly/12 weeks	CSDD
Petersen (2017) [44]	USA	RCT block design	AAT Control	35 26	83.4	N/A	Live Animal–Dog	Single–AAT	Social & Emotional	Active	Social group, no pet	20 min/3× weekly/3 months	CSDD
Pu (2020) [45]	Australia	Pilot RCT	PARO Control	21 22	86.48 85.55	7.71 12.05	Robotic–PARO	Single–Robotic	Social & Emotional	Passive	Usual care, no pet	30 min/2× weekly/12 weeks	CSDD
Robinson (2013) [46]	New Zealand	parallel-group RTC	PARO Control	17 17	55–100	N/A	Robotic–PARO	Single–Robotic	Social & Emotional	Passive	Usual care only	60 min/2× weekly/12 weeks	GDS
Travers (2013) [47]	Australia	parallel-group RTC	AAT Control	27 28	84.9 85.1	58.1 59.8 (3MS)	Live Animal–Dog	Single–AAT	Social & Emotional	Active	Non-pet social games	40–50 min/2× weekly/11 weeks	GDS-SF
Vegue Parra (2021) [16]	Spain	parallel-group RTC	AAT Control	171 163	>65	13.54 13.04	Live Animal–Dog	Single–AAT	Cognitive stimulation	Passive	Usual care, no pet	45 min/once weekly/8 months	CSDD

Abbreviations: RCT: Randomized Controlled Trial; AAT: Animal-Assisted Therapy; PARO: Paro Therapeutic Robot; IEPT: Individualized Emotional & Physical Training; ROT: Reality Orientation Therapy; MMSE: Mini-Mental State Examination; ACE-NZ: Addenbrooke’s Cognitive Examination—New Zealand version; 3MS: Modified Mini-Mental State Examination; GDS: Geriatric Depression Scale; BDI-II: Beck Depression Inventory—Second Edition; CSDD: Cornell Scale for Depression in Dementia.

**Table 2:** Ranking and pairwise comparisons among all intervention groups.

Ranking of Pet-Assisted Interventions			
Live Animal Dog		−1.39 [−2.21; −0.57]	−2.04 [−3.03; −1.04]
−0.83 [−2.06; 0.40]	Robotic PARO	−0.56 [−1.48; 0.37]	
−1.39 [−2.21; −0.57]	−0.56 [1.48; 0.37]	Active Control	
−2.04 [−3.03; −1.04]	−1.21 [−2.79; 0.38]	−0.65 [−1.94; 0.64]	Passive Control

**Table 3:** Ranking and pairwise comparisons of pet-assisted interventions stratified by modality and content.

a. Ranking by modality.						
Combined AAT Gait					−4.55 [−7.15; −1.95]	−4.49 [−6.90; −2.08]
−2.31 [−5.17; 0.55]	Combined AAT IEPT				−1.85 [−3.99; 0.29]	
−2.40 [−5.33; 0.53]	−0.09 [−3.18; 3.00]	Combined AAT ROT			−1.76 [−4.00; 0.47]	
−3.32 [−5.22; −1.42]	−1.01 [−3.33; 1.32]	−0.92 [−3.33; 1.49]	Single AAT		−0.79 [−1.74; 0.15]	−1.56 [−2.58; −0.54]
−3.60 [−5.69; −1.52]	−1.29 [−3.60; 1.01]	−1.20 [−3.60; 1.19]	−0.28 [−1.54; 0.98]	Single Robotic	−0.56 [−1.42; 0.30]	
−4.16 [−6.06; −2.26]	−1.85 [−3.99; 0.29]	−1.76 [−4.00; 0.47]	−0.84 [−1.76; 0.07]	−0.56 [−1.42; 0.30]	Active Control	
−4.82 [−6.69; −2.95]	−2.51 [−5.01; −0.01]	−2.42 [−5.00; 0.16]	−1.50 [−2.48; −0.52]	−1.22 [−2.77; 0.34]	−0.66 [−1.95; 0.63]	Passive Control
b. Ranking by content.						
Physical Functional activity					−4.55 [−7.18; −1.92]	−4.49 [−6.93; −2.05]
−3.06 [−5.25; −0.87]		Cognitive Social			−1.64 [−3.14; −0.14]	−0.65 [−2.83; 1.52]
−3.35 [−5.27; −1.43]		−0.28 [−1.68; 1.11]	Social Emotional		−0.68 [−1.44; 0.09]	−1.83 [−3.00; −0.66]
−3.52 [−5.79; −1.24]		−0.45 [−2.25; 1.35]	−0.17 [−1.62; 1.29]	Cognitive stimulation	−0.68 [−1.93; 0.58]	
−4.19 [−6.09; −2.30]		−1.13 [−2.42; 0.16]	−0.84 [−1.57; −0.11]	−0.68 [−1.93; 0.58]	Active Control	
−4.80 [−6.67; −2.93]		−1.73 [−3.20; −0.27]	−1.45 [−2.49; −0.41]	−1.28 [−3.00; 0.43]	−0.61 [−1.78; 0.56]	Passive Control

## Data Availability

The original contributions presented in this study are included in the article/Supplementary Material. Further inquiries can be directed to the corresponding authors.

## Supplementary Materials

The following supporting information can be downloaded at: https://www.mdpi.com/article/10.3390/healthcare14010038/s1, Table S1. PRISMA-NMA checklist, Table S2. Database search results and screening process, Table S3. Reasons for exclusion, Table S4. Risk-of-bias details, Table S5. Inconsistency test outcomes (a–c), Figure S1. Risk-of-bias summary, Figure S2. Pairwise comparisons (primary), Figure S3a,b. Pairwise comparisons by modality and content, Figure S4A–T. Leave-one-out analysis, Figure S4 (Cog). Cognitive-stratified sensitivity analysis, Figure S5. Publication bias (Egger’s test). Refs. [5,6,7,10,11,15,16,35,36,37,38,39,40,41,42,43,44,45,46,47] are cited in supplementary file.

## References

[1] Tang T., Jiang J., Tang X. (2022). Prevalence of Depression among Older Adults Living in Care Homes in China: A Systematic Review and Meta-Analysis. Int. J. Nurs. Stud..

[2] Rivera E., Hirschman K.B., Naylor M.D. (2020). Reported Needs and Depressive Symptoms among Older Adults Entering Long-Term Services and Supports. Innov. Aging.

[3] Wassink-Vossen S., Oude Voshaar R.C., Naarding P., Collard R.M. (2022). Effectiveness of Late-Life Depression Interventions on Functional Limitations: A Systematic Review. Int. J. Ment. Health Nurs..

[4] Forbes M., Lotfaliany M., Mohebbi M., Reynolds C.F., Woods R.L., Orchard S., Chong T., Agustini B., O’Neil A., Ryan J. (2024). Depressive Symptoms and Cognitive Decline in Older Adults. Int. Psychogeriatr..

[5] Ambrosi C., Zaiontz C., Peragine G., Sarchi S., Bona F. (2019). Randomized Controlled Study on the Effectiveness of Animal-Assisted Therapy on Depression, Anxiety, and Illness Perception in Institutionalized Elderly. Psychogeriatrics.

[6] Baek S.M., Lee Y., Sohng K.Y. (2020). The Psychological and Behavioural Effects of an Animal-Assisted Therapy Programme in Korean Older Adults with Dementia. Psychogeriatrics.

[7] Majić T., Gutzmann H., Heinz A., Lang U.E., Rapp M.A. (2013). Animal-Assisted Therapy and Agitation and Depression in Nursing Home Residents with Dementia: A Matched Case-Control Trial. Am. J. Geriatr. Psychiatry.

[8] Jessen J., Cardiello F., Baun M.M. (1996). Avian Companionship in Alleviation of Depression, Loneliness, and Low Morale of Older Adults in Skilled Rehabilitation Units. Psychol. Rep..

[9] Villarreal-Zegarra D., Yllescas-Panta T., Malaquias-Obregon S., Dámaso-Román A., Mayo-Puchoc N. (2024). Effectiveness of Animal-Assisted Therapy and Pet-Robot Interventions in Reducing Depressive Symptoms among Older Adults: A Systematic Review and Meta-Analysis. Complement. Ther. Med..

[10] An H.J., Park S.J. (2021). Effects of Animal-Assisted Therapy on Gait Performance, Respiratory Function, and Psychological Variables in Patients Post-Stroke. Int. J. Environ. Res. Public Health.

[11] Kil T., Yoon K.A., Ryu H., Kim M. (2019). Effect of Group Integrated Intervention Program Combined Animal-Assisted Therapy and Integrated Elderly Play Therapy on Live Alone Elderly. J. Anim. Sci. Technol..

[12] Sbrizzi C., Sapuppo W. (2021). Effects of Pet Therapy in Elderly Patients with Neurocognitive Disorders: A Brief Review. Dement. Geriatr. Cogn. Dis. Extra.

[13] Arsovski D. (2024). The Role of Animal Assisted Therapy in the Rehabilitation of Mental Health Disorders: A Systematic Literature Review. Perspect. Integr. Med..

[14] Zhou Z., Mei H., Li R., Wang C., Fang K., Wang W., Tang Y., Dai Z. (2022). Progresses of Animal Robots: A Historical Review and Perspectiveness. Heliyon.

[15] Chen S.-C., Lin M.-F., Jones C., Chang W.H., Lin S.-H., Chien C.-O., Hsu C.-F., Qiu H.-Y., Moyle W. (2024). Effect of a Group-Based Personal Assistive Robot (Paro) Robot Intervention on Cognitive Function, Autonomic Nervous System Function, and Mental Well-Being in Older Adults with Mild Dementia: A Randomized Controlled Trial. J. Am. Med. Dir. Assoc..

[16] Vegue Parra E., Hernández Garre J.M., Echevarría Pérez P. (2021). Benefits of Dog-Assisted Therapy in Patients with Dementia Residing in Aged Care Centers in Spain. Int. J. Environ. Res. Public Health.

[17] Orr N., Abbott R., Bethel A., Paviour S., Whear R., Garside R., Coon J.T. (2023). What Are the Effects of Animals on the Health and Wellbeing of Residents in Care Homes? A Systematic Review of the Qualitative and Quantitative Evidence. BMC Geriatr..

[18] Bradwell H.L., Edwards K., Shenton D., Winnington R., Thill S., Jones R.B. (2021). User-Centered Design of Companion Robot Pets Involving Care Home Resident-Robot Interactions and Focus Groups with Residents, Staff, and Family: Qualitative Study. JMIR Rehabil. Assist. Technol..

[19] Dias S., Caldwell D.M. (2019). Network Meta-Analysis Explained. Arch. Dis. Child. Fetal Neonatal Ed..

[20] Balduzzi S., Rücker G., Nikolakopoulou A., Papakonstantinou T., Salanti G., Efthimiou O., Schwarzer G. (2023). Netmeta: An R Package for Network Meta-Analysis Using Frequentist Methods. J. Stat. Softw..

[21] Hutton B., Salanti G., Caldwell D.M., Chaimani A., Schmid C.H., Cameron C., Ioannidis J.P., Straus S., Thorlund K., Jansen J.P. (2015). The Prisma Extension Statement for Reporting of Systematic Reviews Incorporating Network Meta-Analyses of Health Care Interventions: Checklist and Explanations. Ann. Intern. Med..

[22] Zhang J., Yuan Y., Chu H. (2016). The Impact of Excluding Trials from Network Meta-Analyses—An Empirical Study. PLoS ONE.

[23] Sterne J.A.C., Savović J., Page M.J., Elbers R.G., Blencowe N.S., Boutron I., Cates C.J., Cheng H.Y., Corbett M.S., Eldridge S.M. (2019). Rob 2: A Revised Tool for Assessing Risk of Bias in Randomised Trials. BMJ.

[24] Greenberg S.A. (2012). The Geriatric Depression Scale (GDS). Best Pract. Nurs. Care Older Adults.

[25] Jackson-Koku G. (2016). Beck Depression Inventory. Occup. Med..

[26] Bech P. (2009). Fifty Years with the Hamilton Scales for Anxiety and Depression: A Tribute to Max Hamilton. Psychother. Psychosom..

[27] Chaimani A., Caldwell D.M., Li T., Higgins J.P., Salanti G. (2019). Chapter 11: Undertaking Network Meta-Analyses. Cochrane Handbook for Systematic Reviews of Interventions.

[28] Deeks J.J., Higgins J.P., Altman D.G., Group C.S.M. (2019). Analysing Data and Undertaking Meta-Analyses. Cochrane Handbook for Systematic Reviews of Interventions.

[29] Higgins J.P., Eldridge S., Li T. (2019). Including Variants on Randomized Trials. Cochrane Handbook for Systematic Reviews of Interventions.

[30] Page M.J., Higgins J.P., Sterne J.A. (2019). Assessing Risk of Bias Due to Missing Results in a Synthesis. Cochrane Handbook for Systematic Reviews of Interventions.

[31] Borenstein M., Hedges L.V., Higgins J.P., Rothstein H.R. (2009). Fixed-Effect Versus Random-Effects Models. Introd. Meta-Anal..

[32] Owen R.K., Bradbury N., Xin Y., Cooper N., Sutton A. (2019). Metainsight: An Interactive Web-Based Tool for Analyzing, Interrogating, and Visualizing Network Meta-Analyses Using R-Shiny and Netmeta. Res. Synth. Methods.

[33] Becker L.A. (2000). Effect Size (ES). https://www.uv.es/friasnav/EffectSizeBecker.pdf.

[34] Arevalo-Rodriguez I., Smailagic N., i Figuls M.R., Ciapponi A., Sanchez-Perez E., Giannakou A., Pedraza O.L., Cosp X.B., Cullum S. (2015). Mini-Mental State Examination (MMSE) for the Detection of Alzheimer’s Disease and Other Dementias in People with Mild Cognitive Impairment (MCI). Cochrane Database Syst. Rev..

[35] Bono A., Benvenuti C., Buzzi M., Ciatti R., Chiarelli V., Chiambretto P., Morelli C., Pinciroli M., Pini A., Prestigiacomo T. (2015). Effects of Animal Assisted Therapy (Aat) Carried out with Dogs on the Evolution of Mild Cognitive Impairment. G. Gerontol..

[36] Friedmann E., Galik E., Thomas S.A., Hall P.S., Chung S.Y., McCune S. (2015). Evaluation of a Pet-Assisted Living Intervention for Improving Functional Status in Assisted Living Residents with Mild to Moderate Cognitive Impairment: A Pilot Study. Am. J. Alzheimer’s Dis. Other Dement..

[37] Jøranson N., Pedersen I., Rokstad A.M.M., Ihlebaek C. (2015). Effects on Symptoms of Agitation and Depression in Persons with Dementia Participating in Robot-Assisted Activity: A Cluster-Randomized Controlled Trial. J. Am. Med. Dir. Assoc..

[38] Kil T., Kim H.-m., Kim M. (2019). The Effectiveness of Group Combined Intervention Using Animal-Assisted Therapy and Integrated Elderly Play Therapy. J. Anim. Sci. Technol..

[39] Liang A., Piroth I., Robinson H., MacDonald B., Fisher M., Nater U.M., Skoluda N., Broadbent E. (2017). A Pilot Randomized Trial of a Companion Robot for People with Dementia Living in the Community. J. Am. Med. Dir. Assoc..

[40] Menna L.F., Santaniello A., Gerardi F., Sansone M., Di Maggio A., Di Palma A., Perruolo G., D’Esposito V., Formisano P. (2019). Efficacy of Animal-Assisted Therapy Adapted to Reality Orientation Therapy: Measurement of Salivary Cortisol. Psychogeriatrics.

[41] Moretti F., De Ronchi D., Bernabei V., Marchetti L., Ferrari B., Forlani C., Negretti F., Sacchetti C., Atti A.R. (2011). Pet Therapy in Elderly Patients with Mental Illness. Psychogeriatrics.

[42] Moyle W., Cooke M., Beattie E., Jones C., Klein B., Cook G., Gray C. (2013). Exploring the Effect of Companion Robots on Emotional Expression in Older Adults with Dementia: A Pilot Randomized Controlled Trial. J. Gerontol. Nurs..

[43] Olsen C., Pedersen I., Bergland A., Enders-Slegers M.J., Patil G., Ihlebæk C. (2016). Effect of Animal-Assisted Interventions on Depression, Agitation and Quality of Life in Nursing Home Residents Suffering from Cognitive Impairment or Dementia: A Cluster Randomized Controlled Trial. Int. J. Geriatr. Psychiatry.

[44] Petersen S., Houston S., Qin H., Tague C., Studley J. (2016). The Utilization of Robotic Pets in Dementia Care. J. Alzheimer’s Dis..

[45] Pu L., Moyle W., Jones C., Todorovic M. (2020). The Effect of Using Paro for People Living with Dementia and Chronic Pain: A Pilot Randomized Controlled Trial. J. Am. Med. Dir. Assoc..

[46] Robinson H., MacDonald B., Kerse N., Broadbent E. (2013). The Psychosocial Effects of a Companion Robot: A Randomized Controlled Trial. J. Am. Med. Dir. Assoc..

[47] Travers C., Perkins J., Rand J., Bartlett H., Morton J. (2013). An Evaluation of Dog-Assisted Therapy for Residents of Aged Care Facilities with Dementia. Anthrozoös.

[48] Borgi M., Collacchi B., Giuliani A., Cirulli F. (2020). Dog Visiting Programs for Managing Depressive Symptoms in Older Adults: A Meta-Analysis. Gerontologist.

[49] Chang S.J., Lee J., An H., Hong W.H., Lee J.Y. (2021). Animal-Assisted Therapy as an Intervention for Older Adults: A Systematic Review and Meta-Analysis to Guide Evidence-Based Practice. Worldviews Evid.-Based Nurs..

[50] Uvnäs-Moberg K., Gross M.M., Calleja-Agius J., Turner J.D. (2024). The Yin and Yang of the Oxytocin and Stress Systems: Opposites, yet Interdependent and Intertwined Determinants of Lifelong Health Trajectories. Front. Endocrinol..

[51] Flores-García M., Rizzo A., Garçon-Poca M.Z., Fernández-Dueñas V., Bonaventura J. (2023). Converging Circuits between Pain and Depression: The Ventral Tegmental Area as a Therapeutic Hub. Front. Pharmacol..

[52] Sanaeifar F., Pourranjbar S., Pourranjbar M., Ramezani S., Mehr S.R., Wadan A.S., Khazeifard F. (2024). Beneficial Effects of Physical Exercise on Cognitive-Behavioral Impairments and Brain-Derived Neurotrophic Factor Alteration in the Limbic System Induced by Neurodegeneration. Exp. Gerontol..

