# Supplementary material for: From Dogs to Robots: Pet-Assisted Interventions for Depression in Older Adults—A Network Meta-Analysis of Randomized Controlled Trials
Source: Healthcare (Basel). 2025 Dec 23;14(1):38. doi: 10.3390/healthcare14010038 (PMC12786183; doi:10.3390/healthcare14010038)
Supplement: Supplementary file 1 [file healthcare-14-00038-s001.zip › healthcare-3947892-supplementary.pdf]

**Table S1 - PRISMA for network meta-analysis checklist**

| Section and Topic | # | Checklist item | Location |
|---|---|---|---|
| **Title** | | | |
| Title | 1 | Identify the report as a systematic review incorporating a network meta-analysis (or related form of meta-analysis). | Title |
| **Abstract** | | | |
| Structured summary | 2 | Provide a structured summary including, as applicable: Background: main objectives / Methods: data sources; study eligibility criteria, participants, and interventions; study appraisal; and synthesis methods, such as network meta-analysis. / Results: number of studies and participants identified; summary estimates with corresponding confidence/credible intervals; treatment rankings may also be discussed. Authors may choose to summarize pairwise comparisons against a chosen treatment included in their analyses for brevity. / Discussion/conclusions: limitations; conclusions and implications of findings. / Other: primary source of funding; systematic review registration number with registry name. | Abstract |
| **Introduction** | | | |
| Rationale | 3 | Describe the rationale for the review in the context of what is already known, including mention of why a network meta-analysis has been conducted. | Introduction / 1st and 2nd paragraph |
| Objectives | 4 | Provide an explicit statement of questions being addressed, with reference to participants, interventions, comparisons, outcomes, and study design (PICOS). | Introduction, last paragraph |
| **Methods** | | | |
| Protocol & registration | 5 | Indicate whether a review protocol exists and where it can be accessed; and, if available, provide registration information, including registration number. | Materials and Methods/ 1st paragrph |
| Eligibility criteria | 6 | Specify study characteristics (e.g., PICOS, length of follow-up) and report characteristics (e.g., years considered, language, publication status) used as criteria for eligibility, giving rationale. Clearly describe eligible treatments included in the treatment network, and note whether any have been clustered or merged into the same node (with justification). | Materials and Methods/ Inculusion and exculusion |
| Information sources | 7 | Describe all information sources (e.g., databases with dates of coverage, contact with study authors) in the search and date last searched. | Materials and Methods/ Table S2 |
| Search | 8 | Present full electronic search strategy for at least one database, including any limits used, such that it could be repeated. | Materials and Methods/ Table S2 |
| Study selection | 9 | State the process for selecting studies (i.e., screening, eligibility, included in systematic review, and, if applicable, included in the meta-analysis). | Materials and Methods / Study Identification / Inculusion and exculusion criteria |
| Data collection | 10 | Describe method of data extraction from reports (e.g., piloted forms, independently, in duplicate) and any processes for obtaining and confirming data. | Materials and Methods / Data extraction |
| Data items | 11 | List and define all variables for which data were sought (e.g., PICOS, funding sources) and any assumptions and simplifications made. | Materials and Methods / Data extraction and conversion |
| Network geometry | S1 | Describe methods used to explore the geometry of the treatment network under study and potential biases related to it. This should include how the evidence base has been graphically summarized for presentation, and what characteristics were compiled and used to describe the evidence base to readers. | Materials and Methods / Modeling for network meta-analysis |
| Risk of bias within | 12 | Describe methods used for assessing risk of bias of individual studies (including specification of whether this was done at the study or outcome level), and how this information is to be used in any data synthesis. | Materials and Methods / Quality appraisal |
| Summary measures | 13 | State the principal summary measures (e.g., risk ratio, difference in means). Also describe the use of additional summary measures assessed, such as treatment rankings, as well as modified approaches used to present summary findings from meta-analyses. | Materials and Methods / Outcome |

| | | | |
|---|---|---|---|
| Planned methods of analysis | 14 | Describe the methods of handling data and combining results of studies for each network meta-analysis. This should include, but not be limited to: Handling of multi-arm trials; Selection of variance structure; Selection of prior distributions in Bayesian analyses; and Assessment of model fit. | Materials and Methods / Statistical analyses |
| Assessment of inconsistency | S2 | Describe the statistical methods used to evaluate the agreement of direct and indirect evidence in the treatment network(s) studied. Describe efforts taken to address its presence when found. | Materials and Methods / Statistical analyses |
| Risk of bias across | 15 | Specify any assessment of risk of bias that may affect the cumulative evidence. | Materials and Methods / Publication bias |
| Additional analyses | 16 | Describe methods of additional analyses if done, indicating which were pre-specified. This may include, but not be limited to, the following: Sensitivity or subgroup analyses; Meta-regression analyses; Alternative formulations of the treatment network; and Use of alternative prior distributions for Bayesian analyses (if applicable). | Materials and Methods / Sensitivity analyses |
| **Results** | | | |
| Study selection | 17 | Give numbers of studies screened, assessed for eligibility, and included in the review, with reasons for exclusions at each stage, ideally with a flow diagram. | Results / Study identification Figure 1, Table S2, Table S3 |
| Network structure | S3 | Provide a network graph of the included studies to enable visualization of the geometry of the treatment network. | Fugre 2 |
| Network geometry | S4 | Provide a brief overview of characteristics of the treatment network. This may include commentary on the abundance of trials and randomized patients for the different interventions and pairwise comparisons in the network, gaps of evidence in the treatment network, and potential biases reflected by the network structure. | Results / Network model formation / Figure 2 |
| Study characteristics | 18 | For each study, present characteristics for which data were extracted (e.g., study size, PICOS, follow-up period) and provide the citations. | Table 1 |
| Risk of bias within | 19 | Present data on risk of bias of each study and, if available, any outcome level assessment. | Table S4, Figure S1, Methodological quality |
| Results of individual studies | 20 | For all outcomes considered (benefits or harms), present, for each study: (1) simple summary data for each intervention group, and (2) effect estimates and confidence intervals. Modified approaches may be needed to deal with information from larger networks. | Table 1 |
| Synthesis of results | 21 | Present results of each meta-analysis done, including confidence/credible intervals. In larger networks, authors may focus on comparisons versus a particular comparator (e.g. placebo or standard care), with full findings presented in an appendix. League tables and forest plots may be considered to summarize pairwise comparisons. If additional summary measures were explored (such as treatment rankings), these should also be presented. | Outcomes / Figure 3, Figure 4, Figure S2, Figure S3, Table 2 |
| Exploration for inconsistency | S5 | Describe results from investigations of inconsistency. This may include such information as measures of model fit to compare consistency and inconsistency models, P values from statistical tests, or summary of inconsistency estimates from different parts of the treatment network. | Inconsistency test Table S5, Table S6 |
| Risk of bias across | 22 | Present results of any assessment of risk of bias across studies for the evidence base being studied. | Publicaton bias, Figure S6 |
| Additional analyses | 23 | Give results of additional analyses, if done (e.g., sensitivity or subgroup analyses, meta-regression analyses, alternative network geometries studied, alternative choice of prior distributions for Bayesian analyses, and so forth). | Sensitivity analysis / Figure S4, Figre S5 |
| **Discussion** | | | |
| Summary of evidence | 24 | Summarize the main findings, including the strength of evidence for each main outcome; consider their relevance to key groups. | Discussion Findings and implications |

| | | | |
|---|---|---|---|
| Limitations | 25 | Discuss limitations at study and outcome level (e.g., risk of bias), and at review level (e.g., incomplete retrieval of identified research, reporting bias). Comment on the validity of the assumptions, such as transitivity and consistency. Comment on any concerns regarding network geometry (e.g., avoidance of certain comparisons). | Discussion Linitations |
| Conclusions | 26 | Provide a general interpretation of the results in the context of other evidence, and implications for future research. | Conclusion |
| **Funding** | | | |
| Funding | 27 | Describe sources of funding for the systematic review and other support (e.g., supply of data); role of funders for the systematic review. This should also include information regarding whether funding has been received from manufacturers of treatments in the network and/or whether some of the authors are content experts with professional conflicts of interest that could affect use of treatments in the network. | Funding |

**Table S2 - Keywords and search results in different databases** ***1175***

| Database | Keyword | Date | Results |
| --- | --- | --- | --- |
| PubMed | ("pet therapy" OR "animal-assisted intervention" OR "animal-assisted activities" OR "animal-assisted therapy" OR "AAT") AND depression | 2025.08.29 | 232 |
| Embase | ("pet therapy" OR "animal-assisted intervention" OR "animal-assisted activities" OR "animal-assisted therapy" OR "AAT") AND depression | 2025.08.29 | 329 |
| Cochrane CENTRAL | ("pet therapy" OR "animal-assisted intervention" OR "animal-assisted activities" OR "animal-assisted therapy" OR "AAT") AND depression | 2025.08.29 | 109 |
| Web of Science | ("pet therapy" OR "animal-assisted intervention" OR "animal-assisted activities" OR "animal-assisted therapy" OR "AAT") AND depression | 2025.08.29 | 314 |
| | | | 984 |

| Database | Keyword | Date | Results |
| --- | --- | --- | --- |
| PubMed | ("pet therapy" OR "animal-assisted intervention" OR "animal-assisted activities" OR "animal-assisted therapy" OR "AAT") AND "the elderly" AND depression | 2025.08.29 | 11 |
| Embase | ("pet therapy" OR "animal-assisted intervention" OR "animal-assisted activities" OR "animal-assisted therapy" OR "AAT") AND "the elderly" AND depression | 2025.08.29 | 11 |
| Cochrane CENTRAL | ("pet therapy" OR "animal-assisted intervention" OR "animal-assisted activities" OR "animal-assisted therapy" OR "AAT") AND "the elderly" AND depression | 2025.08.29 | 5 |
| Web of Science | ("pet therapy" OR "animal-assisted intervention" OR "animal-assisted activities" OR "animal-assisted therapy" OR "AAT") AND "the elderly" AND depression | 2025.08.29 | 19 |
| | | | 46 |

| Database | Keyword | Date | Results |
| --- | --- | --- | --- |
| PubMed | ("pet therapy" OR "animal-assisted intervention" OR "animal-assisted activities" OR "animal-assisted therapy" OR "AAT") AND (depression) AND (elderly OR "older adults" OR aged OR geriatric) AND ("randomized controlled trial" OR RCT) | 2025.08.29 | 28 |
| Embase | ("pet therapy" OR "animal-assisted intervention" OR "animal-assisted activities" OR "animal-assisted therapy" OR "AAT") AND (depression) AND (elderly OR "older adults" OR aged OR geriatric) AND ("randomized controlled trial" OR RCT) | 2025.08.29 | 52 |
| Cochrane CENTRAL | ("pet therapy" OR "animal-assisted intervention" OR "animal-assisted activities" OR "animal-assisted therapy" OR "AAT") AND (depression) AND (elderly OR "older adults" OR aged OR geriatric) AND ("randomized controlled trial" OR RCT) | 2025.08.29 | 24 |
| Web of Science | ("pet therapy" OR "animal-assisted intervention" OR "animal-assisted activities" OR "animal-assisted therapy" OR "AAT") AND (depression) AND (elderly OR "older adults" OR aged OR geriatric) AND ("randomized controlled trial" OR RCT) | 2025.08.29 | 14 |
| | | | 118 |

| Database | Keyword | Date | Results |
| --- | --- | --- | --- |
| PubMed | ("PARO" OR "PARO robotic pet" OR "PARO robot therapy" OR "PARO robot intervention" AND (depression) AND (elderly OR "older adults" OR aged OR geriatric) AND ("randomized controlled trial" OR RCT) | 2025.08.29 | 10 |
| Embase | ("PARO" OR "PARO robotic pet" OR "PARO robot therapy" OR "PARO robot intervention" AND (depression) AND (elderly OR "older adults" OR aged OR geriatric) AND ("randomized controlled trial" OR RCT) | 2025.08.29 | 17 |
| Cochrane CENTRAL | ("PARO" OR "PARO robotic pet" OR "PARO robot therapy" OR "PARO robot intervention" AND (depression) AND (elderly OR "older adults" OR aged OR geriatric) AND ("randomized controlled trial" OR RCT) | 2025.08.29 | 0 |
| Web of Science | ("PARO" OR "PARO robotic pet" OR "PARO robot therapy" OR "PARO robot intervention" AND (depression) AND (elderly OR "older adults" OR aged OR geriatric) AND ("randomized controlled trial" OR RCT) | 2025.08.29 | 0 |
| | | | 27 |

**Table S3. Reasons for full-text exclusions**

| | Authors & year | Title | Journal/Book | Exclusion reasons |
|---|---|---|---|---|
| 1 | Alegre et al., 2012 | $\alpha$1-Antitrypsin in fibromyalgia: results of a randomized, placebo-controlled, double-blind and crossover pilot trial | Musculoskeletal Care | Unsuitable population |
| 2 | Apóstolo et al., 2019 | Effectiveness of a Combined Intervention on Psychological and Physical Capacities of Frail Older Adults: A Cluster Randomized Controlled Trial | International Journal of Environmental Research and Public Health | Unrelated |
| 3 | Chen et al., 2021 | Animal-Assisted Therapy in Middle-Aged and Older Patients With Schizophrenia: A Randomized Controlled Trial. | Front Psychiatry | Unsuitable population |
| 4 | Cole et al., 2007 | Animal-assisted therapy in patients hospitalized with heart failure | American Journal of Critical Care | Unsuitable population |
| 5 | Colombo et al., 2006 | Pet therapy and institutionalized elderly: a study on 144 cognitively unimpaired subjects | Archives of Gerontology and Geriatrics | Unsuitable population |
| 6 | Friedmann et al., 2015 | Evaluation of a pet-assisted living intervention for improving functional status in assisted living residents with mild to moderate cognitive impairment: a pilot study | American Journal of Alzheimer's Disease and Other Dementias | Unsuitable population |
| 7 | Gebhart et al., 2020 | Distraction-focused interventions on examination stress in nursing students: Effects on psychological stress and biomarker levels. A randomized controlled trial | International Journal of Nursing Practice | Unsuitable population |
| 8 | Gee et al., 2025 | A Pilot Randomized Controlled Trial to Examine the Impact of a Therapy Dog Intervention on Depression, Mood, and Anxiety in Hospitalized Older Adults | Healthcare (Basel) | Non-disaggregated outcome data |
| 9 | Gramaglia et al., 2021 | Non-pharmacological Approaches to Depressed Elderly With No or Mild Cognitive Impairment in Long-Term Care Facilities. A Systematic Review of the Literature | Front Public Health | Review & meta analysis |
| 10 | Grubbs et al., 2016 | A Pilot Study to Assess the Feasibility of Group Exercise and Animal-Assisted Therapy in Older Adults | Journal of Aging and Physical Activity | No relevant outcome data |
| 11 | Hsiao et al., 2006 | Postoperative sedation after major surgery with midazolam or propofol in the ICU: effects on amnesia and anxiety | Acta Anaesthesiologica Taiwanica | Unrelated |
| 12 | Jessen et al., 1996 | Jessen J, Cardiello F, Baun MM. Avian companionship in alleviation of depression, loneliness, and low morale of older adults in skilled rehabilitation units. Psychol Rep. 1996;78(1):339–348. | Psychological Reports | Pet type not eligible |
| 13 | Johnson et al., 2018 | Effects of therapeutic horseback riding on post-traumatic stress disorder in military veterans | Military Medical Research | Unrelated |
| 14 | Kamioka et al., 2014 | Effectiveness of animal-assisted therapy: A systematic review of randomized controlled trials | Complementary Therapies in Medicine | Review & meta analysis |
| 15 | Kelker et al., 2025 | Therapy Dogs for Anxiety in Children in the Emergency Department: A Randomized Clinical Trial | JAMA Network Open | Unsuitable population |
| 16 | Ko et al., 2016 | Effect of Pet Insects on the Psychological Health of Community-Dwelling Elderly People: A Single-Blinded, Randomized, Controlled Trial | Gerontology | Pet type not eligible |
| 17 | Lam et al., 2024 | The effects of a therapy dog intervention on dental fear and anxiety in adult patients undergoing dental procedures: a pilot study | General Dentistry | Unsuitable population |
| 18 | Lisanby et al., 2020 | Neurocognitive Effects of Combined Electroconvulsive Therapy (ECT) and Venlafaxine in Geriatric Depression: Phase 1 of the PRIDE Study | The American Journal of Geriatric Psychiatry | Unrelated |
| 19 | Lisanby et al., 2022 | Longitudinal Neurocognitive Effects of Combined Electroconvulsive Therapy (ECT) and Pharmacotherapy in Major Depressive Disorder in Older Adults: Phase 2 of the PRIDE Study | The American Journal of Geriatric Psychiatry | Unrelated |
| 20 | McCullough et al., 2018 | Measuring the Effects of an Animal-Assisted Intervention for Pediatric Oncology Patients and Their Parents: A Multisite Randomized Controlled Trial [Formula: see text] | Journal of Pediatric Oncology Nursing | Unrelated |
| 21 | Mittly et al., 2024 | The role of dog therapy in clinical recovery and improving quality of life: a randomized, controlled trial | BMC Complementary Medicine and Therapies | Unsuitable population |
| 22 | Mota Pereira et al., 2018 | Pets enhance antidepressant pharmacotherapy effects in patients with treatment resistant major depressive disorder. | Journal of Psychiatric Research | Mean age <65 y (not older adult) |
| 23 | Nasrin et al., 2017 | Effects of Brief Behavioural Activation on Approach and Avoidance Tendencies in Acute Depression: Preliminary Findings | Behavioural and Cognitive Psychotherapy | Unrelated |
| 24 | Olsen et al., 2016 | Effect of animal-assisted activity on balance and quality of life in home-dwelling persons with dementia | Geriatric Nursing | Unrelated |
| 25 | Richerson et al., 2023 | Therapeutic and Economic Benefits of Service Dogs Versus Emotional Support Dogs for Veterans With PTSD | Psychiatric Services | Unrelated |

| | | | | |
|---|---|---|---|---|
| 26 | Sarman et al., 2024 | Goldfish or aquatic turtle? Impact of two animal assisted interventions on children's pain, anxiety, and fear during IV catheterization: A randomized controlled trial | Journal of Pediatric Nursing: Nursing Care of Children and Families | Unrelated |
| 27 | Thodberg et al., 2016 | Therapeutic effects of dog visits in nursing homes for the elderly. | Psychogeriatrics | Incomplete outcome data |
| 28 | Vignolo et al., 2024 | Dog-assisted physiotherapy in amyotrophic lateral sclerosis: a randomized controlled pilot study | European Journal of Physical and Rehabilitation Medicine | Unrelated |
| 29 | Vrijsen et al., 2018 | Cognitive bias modification as an add-on treatment in clinical depression: Results from a placebo-controlled, single-blinded randomized control trial | Journal of Affective Disorders | Irrelevant intervention |
| 30 | Wijker et al., 2020 | Effects of Dog Assisted Therapy for Adults with Autism Spectrum Disorder: An Exploratory Randomized Controlled Trial | Journal of Autism and Developmental Disorders | Unsuitable population |
| 31 | Xue et al., 2007 | Comparisons of the dose-response and recovery time course of vecuronium and atracurium in anesthetized chinese adult patients | Acta Anaesthesiologica Taiwanica | Unsuitable population |
| 32 | Zisselman et al., 1996 | A pet therapy intervention with geriatric psychiatry inpatients | American Journal of Occupational Therapy | Unsuitable population |

**Table S4 - Detailed quality assessment of included studies using Cochrane risk of bias 2 tool**

| First author & Year | Randomization process | Intervention adherence | Missing outcome data | Outcome measurement | Selective reporting | Overall RoB |
|---|---|---|---|---|---|---|
| Ambrosi (2019) | L | L2 | S | S | L | S |
| An (2021) | L | L3 | L | L | L | L |
| Baek (2020) | H | S2 | S | H | L | H |
| Bono (2015) | L | S2 | S | S | L | S |
| Chen (2024) | L | L | L | L | L | L |
| Friedman (2015) | L | S2 | L | L | L | S |
| Jøranson (2015) | L | S2 | L | S | L | S |
| Kil (2019a) | H | S2 | L | S | S | H |
| Kil (2019b) | H | S2 | L | H | S | H |
| Liang (2017) | L | L3 | L | L | L | L |
| Majić (2013) | L | L2 | L | S | L | S |
| Menna (2019) | L | L3 | L | L | L | L |
| Moretti (2010) | H | S2 | L | L | S | S |
| Moyle (2013) | L | L2 | L | S | L | S |
| Olsen (2016) | L | L3 | L | S | L | S |
| Petersen (2017) | S | S2 | L | S | L | S |
| Pu (2020) | L | S2 | L | S | L | S |
| Robinson (2013) | L | L2 | S | S | L | S |
| Travers (2013) | L | S2 | L | L | L | S |
| Vegue Parra (2021) | L | L3 | L | L | L | L |

1 The study employed a waitlist control group design, which resulted in a more balanced comparison among different groups.

2 The differences in protocols among various groups may affect adherence and outcome.

3 Both groups were randomized to receive exercise interventions, and the study design utilized a balanced protocol, which minimized the impact on adherence.

H, high risk of bias; L, low risk of bias; S, some risk of bias.

Table S5. Inconsistency test outcomes for the standardized mean difference (SMD) in reducing depressive symptoms among older adults receiving pet-assisted interventions

| No. | Comparison | No.Studies | NMA | Direct | Indirect | Difference | 95CI_lower | 95CI_upper | p-Value |
|---|---|---|---|---|---|---|---|---|---|
| 1 | Active_Control:Live_Animal_Dog | 8.00 | 1.39 | 1.39 | NA | NA | NA | NA | NA |
| 2 | Active_Control:Passive_Control | 0.00 | -0.65 | NA | -0.65 | NA | NA | NA | NA |
| 3 | Active_Control:Robotic_PARO | 6.00 | 0.56 | 0.56 | NA | NA | NA | NA | NA |
| 4 | Live_Animal_Dog:Passive_Control | 6.00 | -2.04 | -2.04 | NA | NA | NA | NA | NA |
| 5 | Live_Animal_Dog:Robotic_PARO | 0.00 | -0.83 | NA | -0.83 | NA | NA | NA | NA |
| 6 | Robotic_PARO:Passive_Control | 0.00 | -1.21 | NA | -1.21 | NA | NA | NA | NA |

**TableS5a** by pet type

| No. | Comparison | No.Studies | NMA | Direct | Indirect | Difference | 95CI_lower | 95CI_upper | p-Value |
|---|---|---|---|---|---|---|---|---|---|
| 1 | Active_Control:Combined_AAT_Gait | 1.00 | 4.16 | 4.55 | 3.72 | 0.83 | -2.97 | 4.64 | 0.67 |
| 2 | Active_Control:Combined_AAT_IEPT | 1.00 | 1.85 | 1.85 | NA | NA | NA | NA | NA |
| 3 | Active_Control:Combined_AAT_ROT | 1.00 | 1.76 | 1.76 | NA | NA | NA | NA | NA |
| 4 | Active_Control:Passive_Control | 0.00 | -0.66 | NA | -0.66 | NA | NA | NA | NA |
| 5 | Active_Control:Single_AAT | 5.00 | 0.84 | 0.79 | 1.63 | -0.83 | -4.64 | 2.97 | 0.67 |
| 6 | Active_Control:Single_Robotic | 6.00 | 0.56 | 0.56 | NA | NA | NA | NA | NA |
| 7 | Combined_AAT_Gait:Combined_AAT_IEPT | 0.00 | -2.31 | NA | -2.31 | NA | NA | NA | NA |
| 8 | Combined_AAT_Gait:Combined_AAT_ROT | 0.00 | -2.40 | NA | -2.40 | NA | NA | NA | NA |
| 9 | Combined_AAT_Gait:Passive_Control | 1.00 | -4.82 | -4.49 | -5.32 | 0.83 | -2.97 | 4.64 | 0.67 |
| 10 | Combined_AAT_Gait:Single_AAT | 0.00 | -3.32 | NA | -3.32 | NA | NA | NA | NA |
| 11 | Combined_AAT_Gait:Single_Robotic | 0.00 | -3.60 | NA | -3.60 | NA | NA | NA | NA |
| 12 | Combined_AAT_IEPT:Combined_AAT_ROT | 0.00 | -0.09 | NA | -0.09 | NA | NA | NA | NA |
| 13 | Combined_AAT_IEPT:Passive_Control | 0.00 | -2.51 | NA | -2.51 | NA | NA | NA | NA |
| 14 | Combined_AAT_IEPT:Single_AAT | 0.00 | -1.01 | NA | -1.01 | NA | NA | NA | NA |
| 15 | Combined_AAT_IEPT:Single_Robotic | 0.00 | -1.29 | NA | -1.29 | NA | NA | NA | NA |
| 16 | Combined_AAT_ROT:Passive_Control | 0.00 | -2.42 | NA | -2.42 | NA | NA | NA | NA |
| 17 | Combined_AAT_ROT:Single_AAT | 0.00 | -0.92 | NA | -0.92 | NA | NA | NA | NA |
| 18 | Combined_AAT_ROT:Single_Robotic | 0.00 | -1.20 | NA | -1.20 | NA | NA | NA | NA |
| 19 | Single_AAT:Passive_Control | 5.00 | -1.50 | -1.56 | -0.73 | -0.83 | -4.64 | 2.97 | 0.67 |
| 20 | Single_Robotic:Passive_Control | 0.00 | -1.22 | NA | -1.22 | NA | NA | NA | NA |
| 21 | Single_AAT:Single_Robotic | 0.00 | -0.28 | NA | -0.28 | NA | NA | NA | NA |

**Table S5b** by modality

| No. | Comparison | No.Studies | NMA | Direct | Indirect | Difference | 95CI_lower | 95CI_upper | p-Value |
|---|---|---|---|---|---|---|---|---|---|
| 1 | Active_Control:Cognitive_Social | 2.00 | 1.13 | 1.64 | -0.34 | 1.98 | -0.97 | 4.93 | 0.19 |
| 2 | Active_Control:Cognitive_stimulation | 3.00 | 0.68 | 0.68 | NA | NA | NA | NA | NA |
| 3 | Active_Control:Passive_Control | 0.00 | -0.61 | NA | -0.61 | NA | NA | NA | NA |
| 4 | Active_Control:Physical_Functional_activity | 1.00 | 4.19 | 4.55 | 3.80 | 0.75 | -3.05 | 4.55 | 0.70 |
| 5 | Active_Control:Social_Emotional | 8.00 | 0.84 | 0.68 | 2.49 | -1.82 | -4.36 | 0.73 | 0.16 |
| 6 | Cognitive_Social:Cognitive_stimulation | 0.00 | -0.45 | NA | -0.45 | NA | NA | NA | NA |
| 7 | Cognitive_Social:Passive_Control | 1.00 | -1.73 | -0.65 | -2.63 | 1.98 | -0.97 | 4.93 | 0.19 |
| 8 | Cognitive_Social:Physical_Functional_activity | 0.00 | 3.06 | NA | 3.06 | NA | NA | NA | NA |
| 9 | Cognitive_Social:Social_Emotional | 0.00 | -0.28 | NA | -0.28 | NA | NA | NA | NA |
| 10 | Cognitive_stimulation:Passive_Control | 0.00 | -1.28 | NA | -1.28 | NA | NA | NA | NA |
| 11 | Cognitive_stimulation:Physical_Functional_activity | 0.00 | 3.52 | NA | 3.52 | NA | NA | NA | NA |
| 12 | Cognitive_stimulation:Social_Emotional | 0.00 | 0.17 | NA | 0.17 | NA | NA | NA | NA |
| 13 | Physical_Functional_activity:Passive_Control | 1.00 | -4.80 | -4.49 | -5.24 | 0.75 | -3.05 | 4.55 | 0.70 |
| 14 | Social_Emotional:Passive_Control | 4.00 | -1.45 | -1.83 | -0.02 | -1.82 | -4.36 | 0.73 | 0.16 |
| 15 | Physical_Functional_activity:Social_Emotional | 0.00 | -3.35 | NA | -3.35 | NA | NA | NA | NA |

**Table S5c** by content

**Fig S1.** Risk Of Bias Assessment Across 21 Studies (With Percentages)

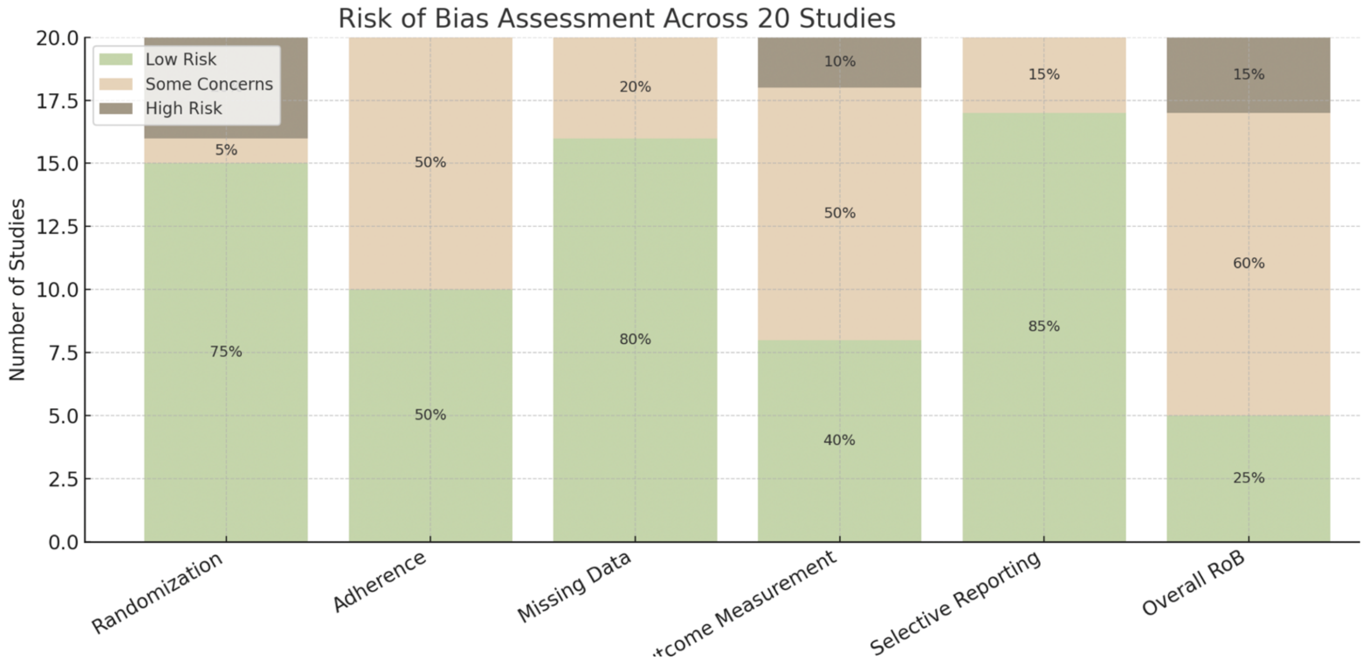


**Figure S1** - Summary of quality assessment for the studies included in the current network meta-analysis using version 2 of the Cochrane risk-of-bias tool for randomized controlled trials.

**Figure S2**

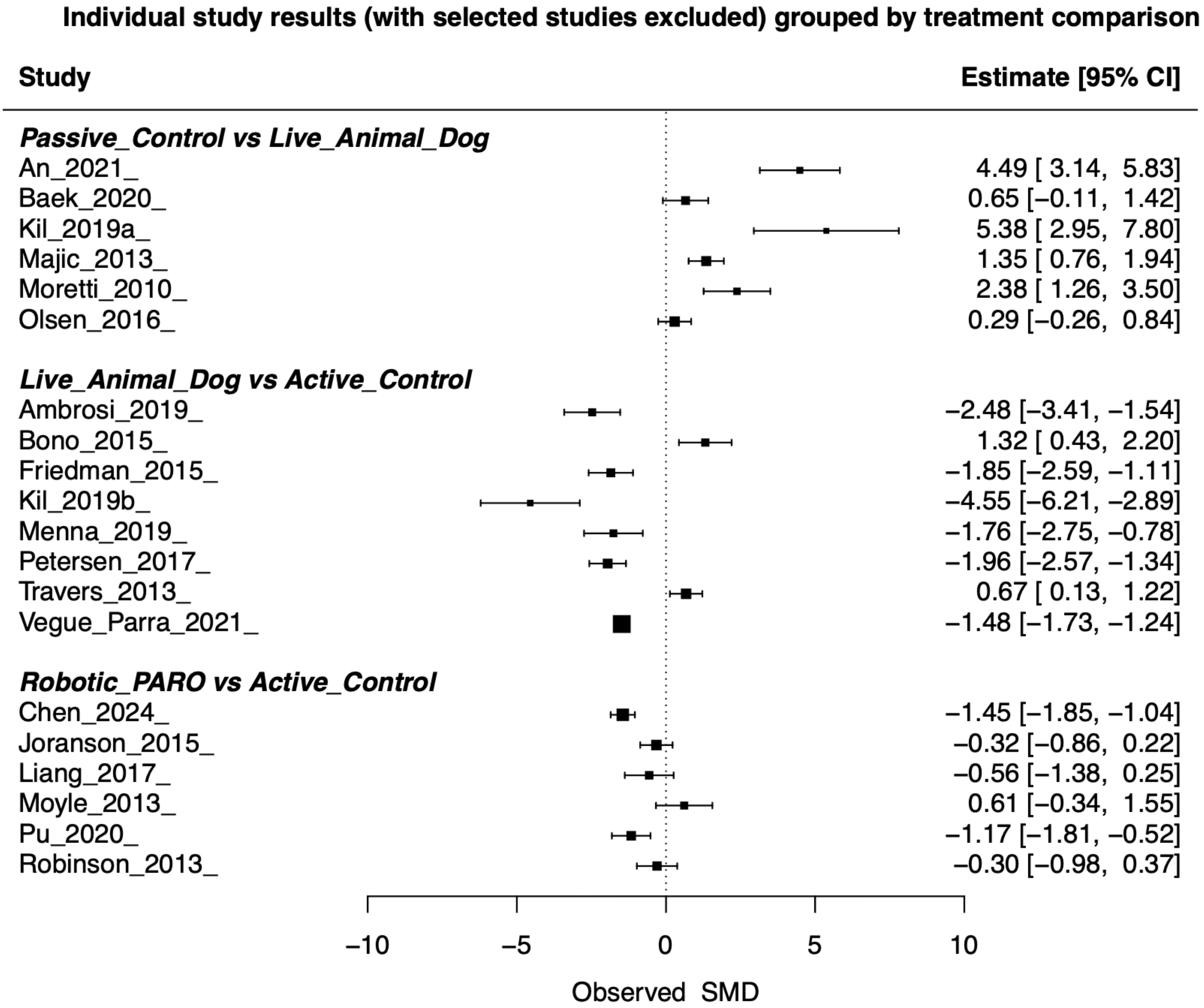


**Fig. S2** – Pairwise comparison of the standardized mean difference (SMD) in depressive symptom scores among different pet-assisted intervention contrasts included in the network meta-analysis.

**Figure S3a**

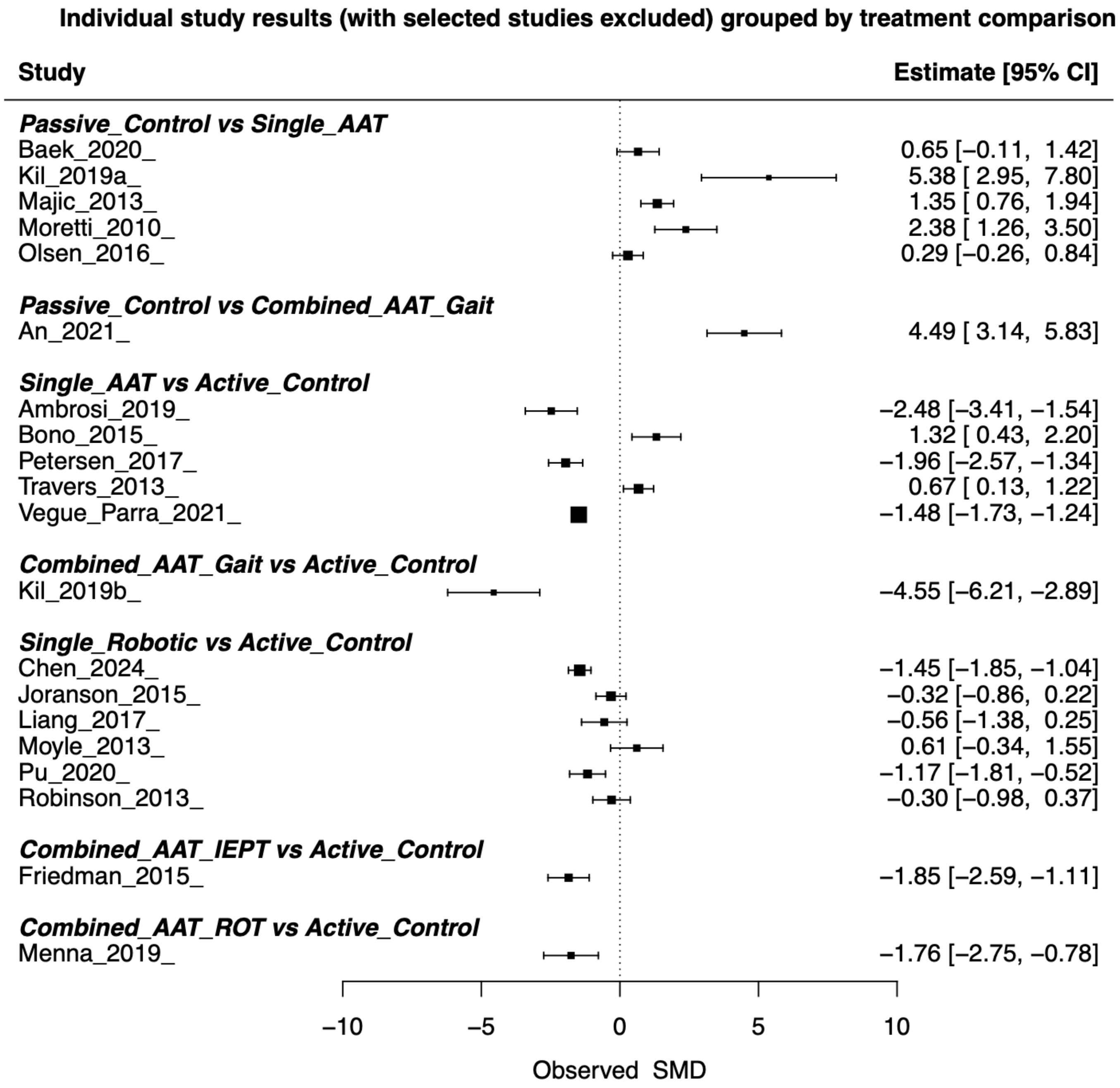


Figure 3Sa – Forest plot of standardized mean differences (SMD) with 95 % confidence intervals for pet-assisted interventions versus control groups in reducing depressive symptoms among older adults, stratified by intervention modality.

**Figure S3b**

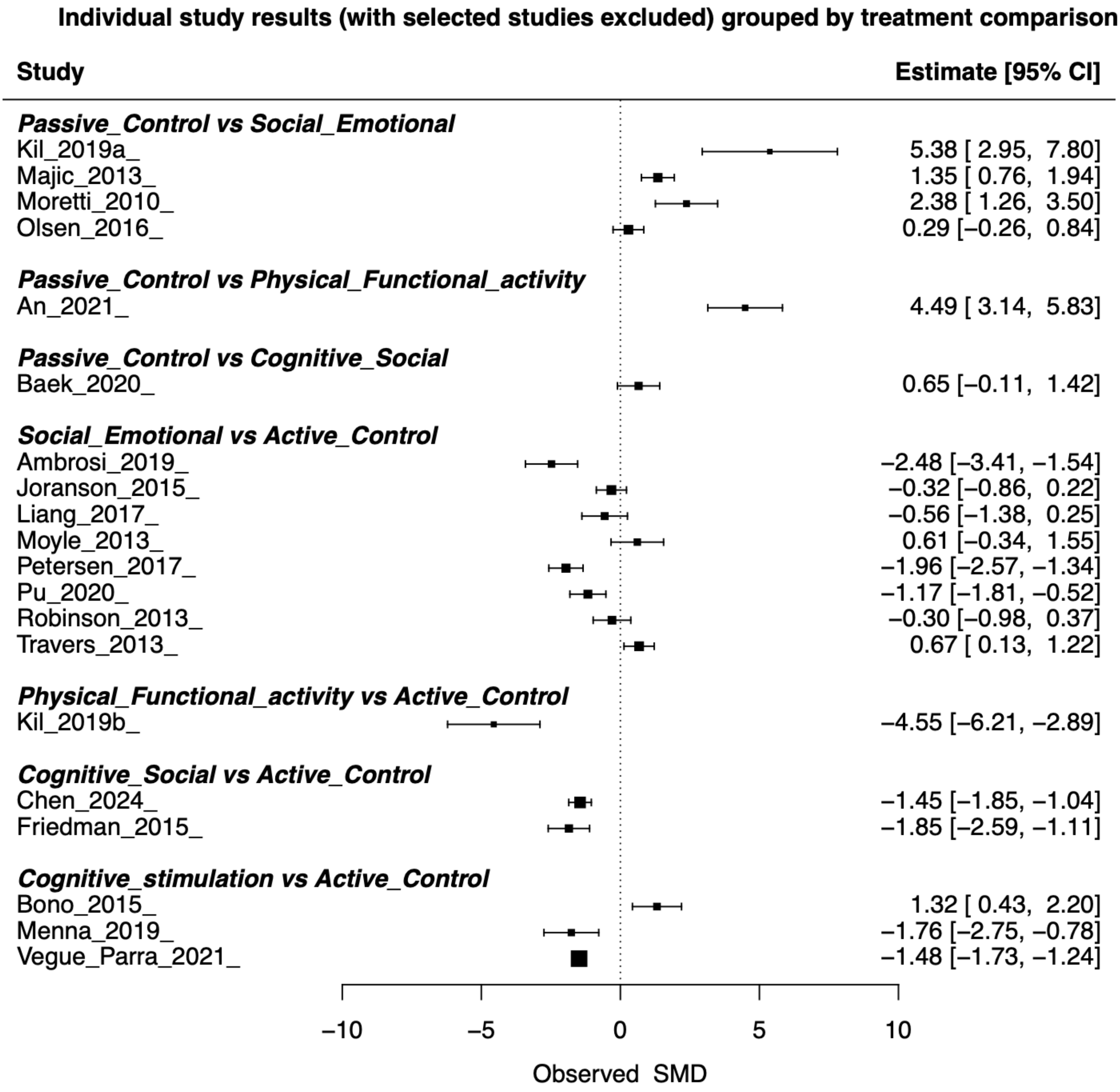


**Figure 3Sb** – Forest plot of standardized mean differences (SMD) with 95 % confidence intervals for pet-assisted interventions versus control groups in reducing depressive symptoms among older adults, stratified by intervention content.

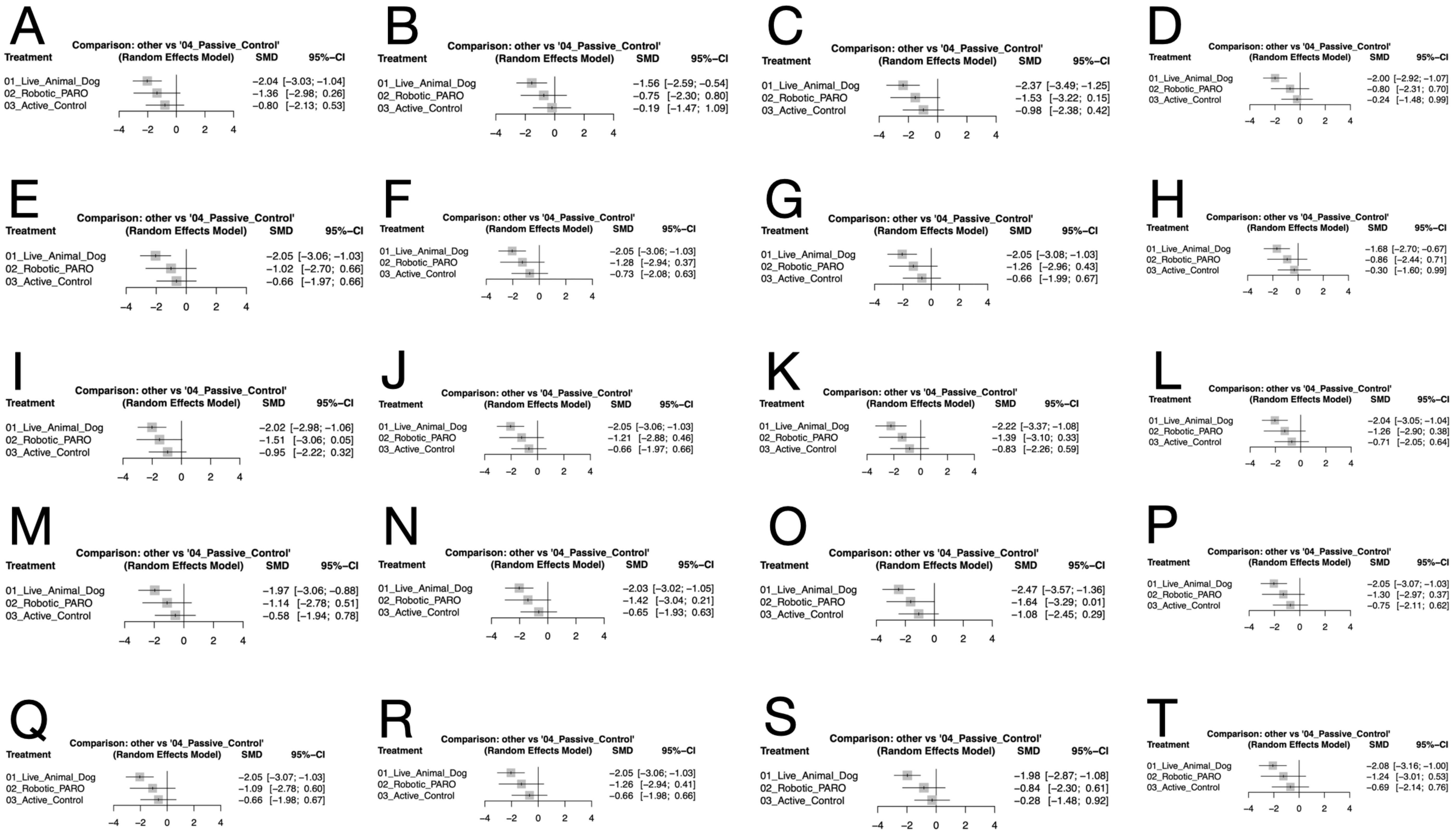


**Studies Excluded One by One (Referenced Alphabetically)**

A. Ambrosi_2019
B. An_2021
C. Baek_2020
D. Bono_2015
E. Chen_2024
F. Friedman_2015
G. Joranson_2015
H. Kil_2019-a
I. Kil_2019-b
J. Liang_2017
K. Majic_2013
L. Menna_2019
M. Moretti_2010
N. Moyle_2013
O. Olsen_2016
P. Petersen_2017
Q. Pu_2020
R. Robinson_2013
S. Travers_2013
T. Vegue_Parra_2021

**Fig. S4** The forest plots display the results of the sensitivity analysis conducted using the one-study removal method, involving 20 studies (labeled A to T). The ranking and clinical significance remain unchanged, indicating that the conclusions of our study are not affected by the inclusion or exclusion of any single study.

Fig-S4

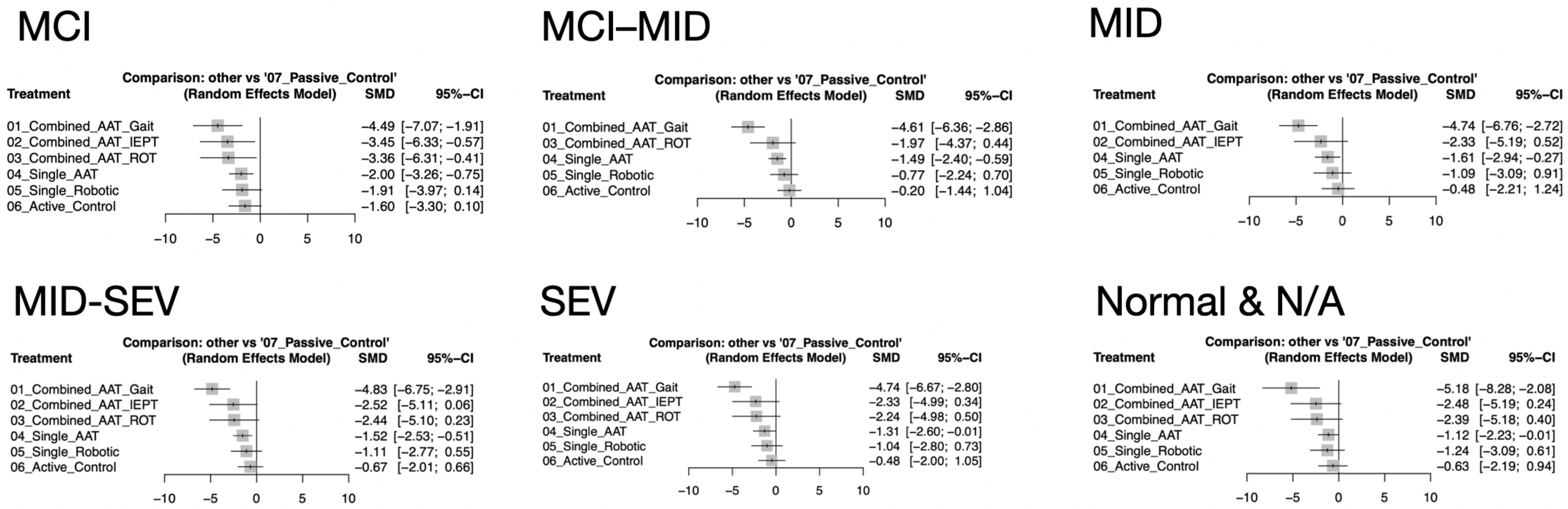


**Studies Excluded by cognitive severity.**

**MCI**
Chen (2024)
Kil (2019b)
Liang (2017)
Olsen (2016)
Ambrosi (2019)

**MCI–MID**
Bono (2015)
Friedman (2015)

**MID**
Baek (2020)
Joranson (2015)
Menna (2019)
Moretti (2010)
Travers (2013)
Vegue Parra (2021)

**MID–SEV**
Pu (2020)

**SEV**
Majic (2013)
Moyle (2013)

**Normal**
An (2021)

**N/A**
Kil (2019a)
Petersen (2017)
Robinson (2013)

**Fig. S4 (Cog).** Sensitivity analysis by cognitive severity. The forest plots present the sensitivity analysis excluding studies according to cognitive function levels (Normal, MCI, MCI–MID, MID, MID–SEV, SEV, and N/A). The pooled estimates and ranking patterns remained stable, indicating that the overall conclusions were not substantially affected by the inclusion of studies with any specific cognitive severity. *Note:* (Cog) denotes sensitivity analysis stratified by cognitive function level.

**Abbreviations:** Normal = normal cognition; MCI = mild cognitive impairment; MCI–MID = mild cognitive impairment to mild dementia; MID = mild dementia; MID–SEV = mild to severe dementia; SEV = severe dementia; N/A = not available or not specified.

# Fig-S4-Cog

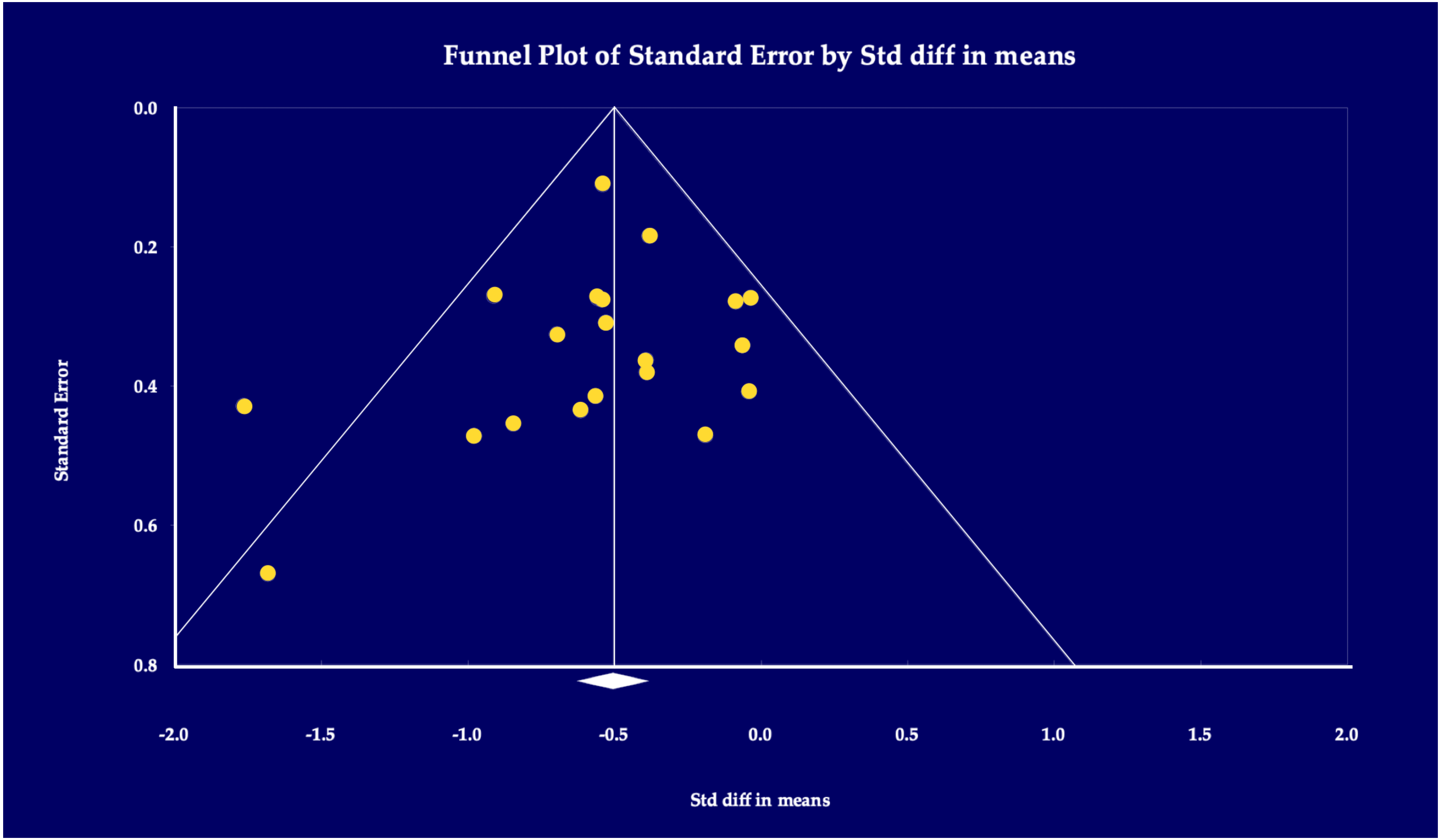


**Figure S5** – Funnel plot of SMDs for depressive symptom reduction. Visual symmetry and Egger's test ($B_0 = -0.48$, 95% CI −1.73 to 0.77; $p = 0.43$) suggest no significant publication bias.
